# Cellular senescence-mediated exacerbation of Duchenne muscular dystrophy

**DOI:** 10.1038/s41598-020-73315-6

**Published:** 2020-10-12

**Authors:** Hidetoshi Sugihara, Naomi Teramoto, Katsuyuki Nakamura, Takanori Shiga, Taku Shirakawa, Masafumi Matsuo, Masashi Ogasawara, Ichizo Nishino, Takashi Matsuwaki, Masugi Nishihara, Keitaro Yamanouchi

**Affiliations:** 1 Department of Veterinary Physiology, Graduate School of Agricultural and Life Sciences, The University of Tokyo, Bunkyo-ku, Tokyo, 113-8657 Japan; 2 Department of Veterinary Pathology, Graduate School of Agricultural and Life Sciences, The University of Tokyo, Bunkyo-ku, Tokyo, 113-8657 Japan; 3 Research Center for Locomotion Biology, Kobe Gakuin University, Nishi, Kobe, 651-2180 Japan; 4 KNC Department of Nucleic Acid Drug Discovery, Faculty of Rehabilitation, Kobe Gakuin University, Nishi, Kobe, 651-2180 Japan; 5 Department of Genome Medicine Development, Medical Genome Center, Kodaira, Tokyo 187-8502 Japan; 6 Department of Neuromuscular Research, National Institute of Neuroscience, National Center of Neurology and Psychiatry (NCNP), Kodaira, Tokyo 187-8502 Japan

**Keywords:** Physiology, Diseases

## Abstract

Duchenne muscular dystrophy (DMD) is a progressive disease characterised by chronic muscle degeneration and inflammation. Our previously established DMD model rats (DMD rats) have a more severe disease phenotype than the broadly used mouse model. We aimed to investigate the role of senescence in DMD using DMD rats and patients. Senescence was induced in satellite cells and mesenchymal progenitor cells, owing to the increased expression of *CDKN2A*, p16- and p19-encoding gene. Genetic ablation of *p16* in DMD rats dramatically restored body weight and muscle strength. Histological analysis showed a reduction of fibrotic and adipose tissues invading skeletal muscle, with increased muscle regeneration. Senolytic drug ABT263 prevented loss of body weight and muscle strength, and increased muscle regeneration in rats even at 8 months—the late stage of DMD. Moreover, senescence markers were highly expressed in the skeletal muscle of DMD patients. In situ hybridization of *CDKN2A* confirmed the expression of it in satellite cells and mesenchymal progenitor cells in patients with DMD. Collectively, these data provide new insights into the integral role of senescence in DMD progression.

## Introduction

Duchenne muscular dystrophy (DMD) is a X-linked muscular dystrophy caused by *DMD* mutations. This gene encodes dystrophin, the structural protein stabilising the plasma membrane of muscle cells, including myofibres and myocardiocytes^1^. *DMD* mutation leads to muscle fragility. Based on the mutation pattern, muscular dystrophies with *DMD* mutations are classified into one of the two types; Duchenne muscular dystrophy, caused by an out-of-frame *DMD* mutation, or Becker muscular dystrophy (BMD), caused by an in-frame *DMD* mutation. DMD is a severe form of muscular dystrophy and affects 1 in 3500 new-born males. DMD occurs as a result of non-sense mutation in *DMD*, and complete loss of dystrophin leads to continuous degeneration of muscle fibres. Due to muscle weakness and atrophy, patients with DMD typically need wheelchairs by the age of 8–14 years, and the condition will ultimately lead to respiratory and heart failure^2^.

Skeletal muscle normally has a high regenerative capacity. Once muscle fibres are damaged, the muscle-specific stem cells, i.e. satellite cells, are activated and proliferate^3^. Some proliferated cells reverse their state back to stem to maintain a satellite cell pool, and the others differentiate into their committed state. The differentiated cells are fused together to form myotubes, whose maturation constitutes newly regenerated myofibres. However, patients with DMD show impaired muscle regeneration^4^. Moreover, progressive fatty and fibrous tissue deposition is observed as the disease advances. Both adipose and fibrous tissues impair skeletal muscle function^5,6^. Recent studies have identified that both adipose and fibrous tissues in skeletal muscle are derived from mesenchymal progenitor cells (MPCs), which reside in the interstitial spaces of skeletal muscle^7–9^. Acute muscle damage stimulates the proliferation of MPCs, which promotes satellite cell differentiation and muscle regeneration through paracrine factors^7^. However, under pathological conditions, MPCs differentiate into adipose and fibrous tissues and inhibit, rather than support, muscle regeneration^8,9^.

Senescence is a state of permanent cell cycle arrest induced by various types of stress, including oxidative stress^10,11^. Although senescence was originally regarded as a process that only occurs in cultured cells, recent studies have shown the appearance of senescent cells in vivo^12^. Senescent cells are distinguished from other non-proliferating cells by several markers, such as the expression of anti-proliferative molecules (e.g. p16^INK4a^, ARF and p21)^13,14^ and the high lysosomal activity of β-galactosidase, termed senescence-associated β-galactosidase (SA-βGal) activity. Both p16 and ARF proteins are encoded by *CDKN2A*. *CDKN2A* is composed of 3 exons (exon1α, exon2, and exon3) whose transcripts generate p16, while the transcript of ARF originates from an additional exon called exon1β and is spliced to the common exon2, leading to the formation of an alternative reading frame^15^. Therefore, p16 and ARF share no amino acid homology although they share exon2 and exon3. In humans, ARF is translated into 14 kDa, called p14^ARF^, while in the case of mice and rats, it is 19 kDa, called p19^ARF^^15^.

Senescent cells cease the cell cycle, and secrete various cytokines, such as interleukin (IL)-6 and transforming growth factor (TGF)-β; this is referred to as the senescence associated secretory phenotype (SASP)^16,17^. SASP is reported not only in mice, but also in rats and humans^14,18^. Senescent cells are reportedly involved in the exacerbation of various diseases in mice models, such as idiopathic pulmonary disease^19^, hepatic steatosis^20^, atherosclerosis^21^, and others^22,23^. Notably in skeletal muscle, senescence in satellite cells in aged mouse skeletal muscle causes cell cycle arrest, resulting in a decrease in the number of satellite cells and decline in regenerative potential in mice^23^. In addition to satellite cells, we previously found the presence of senescent MPCs in aged rat skeletal muscle. SASP of senescent MPCs abrogated myoblast fusion, suggesting an inhibitory role in muscle regeneration in rat^18^.

Persistent muscle damage causes chronic inflammation^4^; this is observed after the increase in skeletal muscle oxidative stress in patients with DMD^24^. As oxidative stress is a cause of senescence, it is plausible that senescence is induced in the skeletal muscle of patients with DMD. To test this hypothesis, we used rats previously generated by our group that carry an out-of-frame mutation in *Dmd* (DMD rats). The most commonly used DMD model animals are mdx mice, which have an out-of-frame mutation in *Dmd*. However, mdx mice do not present a severe disease phenotype, indicative of late stage DMD in humans, and show no, or very little, fibrosis or adipogenesis in their skeletal muscle^25^. Our previous research showed that F0 DMD rats exhibited a more severe phenotype than mdx mice, and represented fibrosis from as early as 12 weeks-old^26^. However, the detailed disease progression of DMD rats is still unknown. In this study, we aimed to investigate the role of senescence in DMD using DMD rats and patients. Elucidating the involvement of senescence in disease progression could potentially serve as a novel therapeutic target for DMD treatment.

## Results

### Exacerbation of dystrophic phenotype in DMD rats

**Figure 1:**
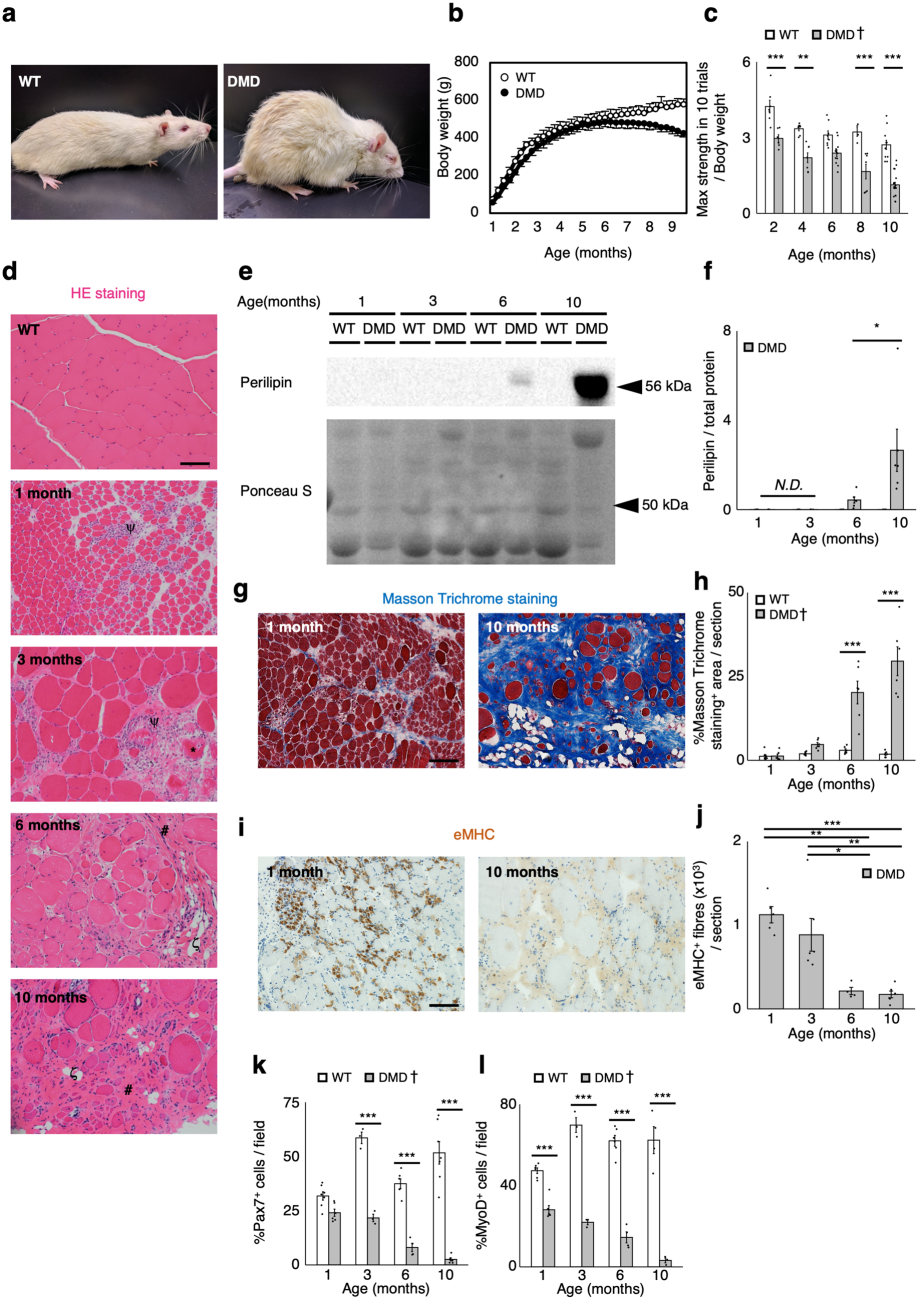
Progressive exacerbation of muscular dystrophy in DMD rats. (**a**) Representative images of 6-month-old WT (left panel) and DMD rats (right panel). (**b**) Body weight comparison of WT and DMD rats of 1–9 months of age (n = 6). (**c**) Quantification of maximum muscle strength by grip test at the indicated ages of WT and DMD rats (2 months: WT: n = 7, DMD: n = 6, 4 months: WT: n = 7, DMD: n = 7, 6 months: WT: n = 8, DMD: n = 12, 8 months: WT: n = 4, DMD: n = 7, 10 months: WT: n = 11, DMD: n = 21). (**d**) Representative HE stains of TA muscle sections from 6-month-old WT rats and 1- to 10-month-old DMD rats. Scale bar = 100 μm. The following symbols, ψ, *, #, and ζ, indicate inflammatory cell infiltration, necrotic myofibres, fibrosis, and adipogenesis, respectively. (**e**) Immunoblotting analysis of perilipin expression in WT and DMD rats. Full-length blots are presented in Supplementary Figure 6a. (**f**) Quantification of perilipin protein expression (n = 6, each). (**g**) Representative Masson Trichrome stains of TA muscle sections from 1- to 10-month-old WT and DMD rats. Scale bar = 100 μm. (**h**) Quantification of Masson Trichrome staining positive area per section (n = 6, each). (**i**) Immunohistochemical analysis of eMHC in TA muscle sections from WT and DMD rats. Scale bar = 100 μm. (**j**) Quantification of eMHC positive fibres per section (n = 6, each). (**k**,**l**) Quantification of (**k**) Pax7^+^ cells and (**l**) MyoD^+^ cells of skeletal muscle primary cells from WT and DMD rats (n = 6, each). Data are expressed as mean ± SEM except for (**b**), which is expressed as mean ± SD, and were compared by Tukey Kramer’s test. Different letters indicate statistically significant differences (p < 0.05). Progressive decrease of both Pax7^+^ and MyoD^+^ cells was observed in DMD rats. Significant decrease of the number of MyoD^+^ cells was observed in DMD rats from 1 month compared to WT, while from 3 months about the number of Pax7^+^ cells. For (**c**), (**h**), (**k**) and (**l**), the result of statistical comparison only between the genotypes at each indicated ages was displayed. When a significant age-related difference was observed by the Tukey–Kramer’s test, the † mark was added beside the legend of the graph. *p < 0.05. **p < 0.01. ***p < 0.001. *N.D.* not detected.

All the animal experiments were performed using male rats. We previously used the CRISPR/Cas system to generate various strains of rats carrying out-of-frame mutations in *Dmd*^26^ in exon3 and exon16. One of the rats (see mutation pattern in Supplementary Figure 1a,b) was selected and used to establish a strain of DMD model rats (DMD rats). Immunoblot analysis confirmed the lack of dystrophin in DMD rats (Supplementary Figure 1c). Progressive exacerbation of the dystrophic phenotype was observed in DMD rats. They showed severe phenotypic traits including rough coats and kyphosis at 6 months (Fig. 1a). Weekly measurements of body weight showed the progressive decrease in body mass in DMD rats after the age of 6 months (Fig. 1b). The grip test analysis showed the functional decline in muscle strength as early as at 1 month, and a further decline was observed until 10 months of age (Fig. 1c). To assess the histopathology of the skeletal muscle, we performed HE staining of tibialis anterior (TA) muscles in DMD rats between 1 and 10 months of age (Fig. 1d). Muscle necrosis and inflammation were observed in 1-month-old rats, and muscle damage markers—creatine kinase and urine titin—decreased with age while these markers were higher until 6 months of age when compared to WT (Supplementary Figure 1d,e). No signal of perilipin was detected in WT rat skeletal muscle by the western blot analysis. On the other hand, increased adipogenesis (Fig. 1e,f; Supplementary Figure 6a) and fibrosis (Fig. 1g,h) were observed in 6-month-old DMD rats, and ultimately resulted in the replacement of skeletal muscle fibres with adipose and fibrotic tissues at 10 months, as would be observed in the late stage of human patients with DMD^4,27^. Progressive fibrosis was also evident in the diaphragm (Supplementary Figure 2a), although the respiratory function was maintained at the same level as in wild-type (WT) rats even at 10 months (Supplementary Figure 2b).

Immunohistochemical analysis of eMHC, which is a marker of regenerating muscle fibre, revealed the progressive decrease in the number of eMHC^+^ myofibres per section from 6 months (Fig. 1i,j). Since there is no injury on muscles of WT, we could not detect any eMHC^+^ fibres. Also, we found the progressive decrease of total number of myofibres and the ratio of eMHC^+^ myofibres with respect to the total number of myofibres (Supplementary Figure 1f,g), suggesting the progressive impairment of muscle regeneration capacity in DMD rat skeletal muscle. As satellite cells are responsible for skeletal muscle regeneration^28,29^, we performed immunocytochemical analyses of Pax7 and MyoD, markers of satellite cells, in WT and DMD rat skeletal muscle primary cells. The quantitative analyses revealed that the proportion of both Pax7 and MyoD positive cells decreased dramatically from 3 months of age in DMD rat primary cells (Fig. 1k,l), suggesting the progressive decrease in the number of satellite cells in skeletal muscle of DMD rats.

### Appearance of senescent cells in DMD rat skeletal muscle

**Figure 2:**
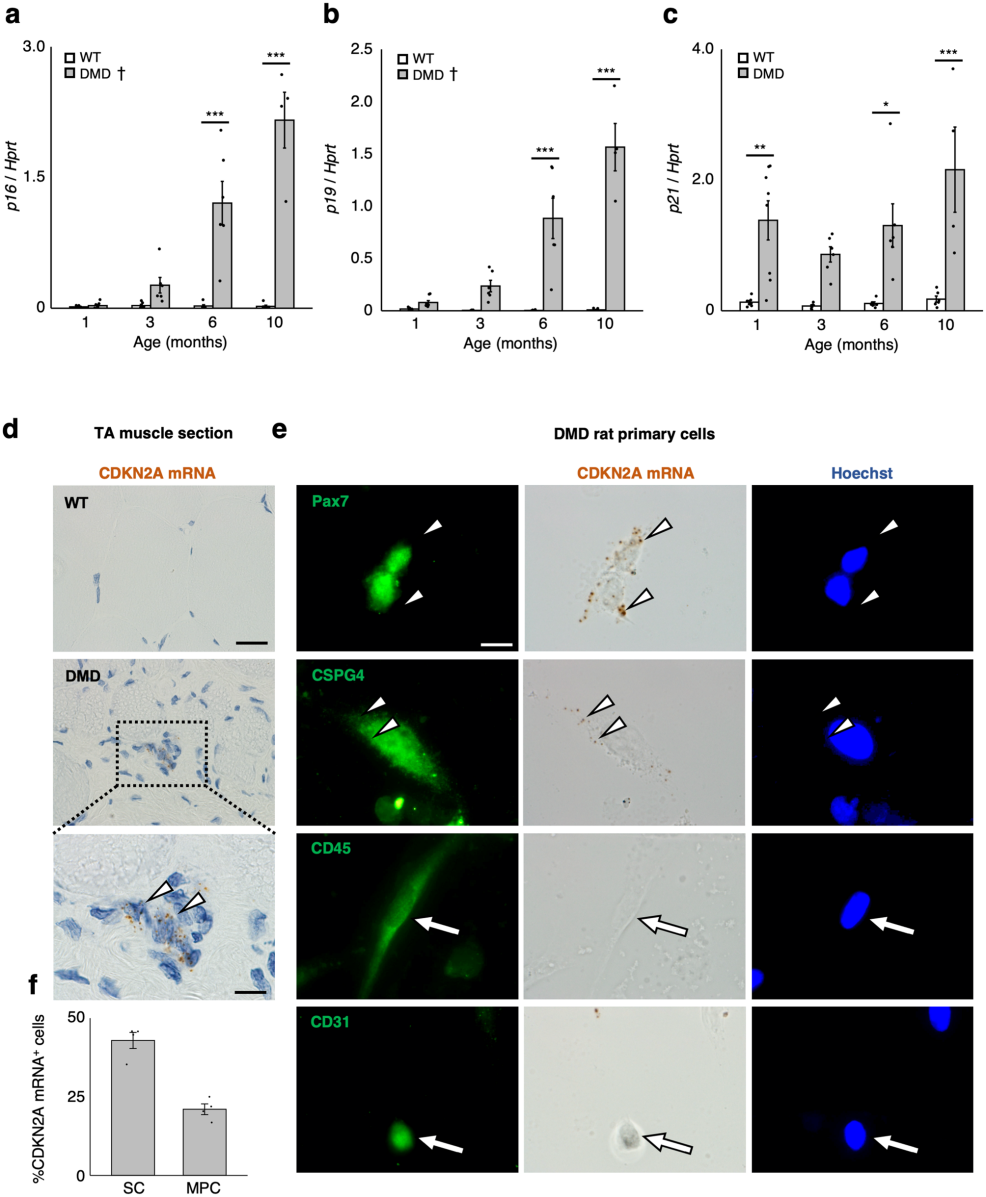
Senescent satellite cells and mesenchymal progenitor cells were present in DMD rats. (**a**–**c**) Quantification of mRNA levels of senescence markers (p16, p19, and p21) in WT and DMD rat TA muscles (n = 6, each). (**d**) In situ hybridisation of CDKN2A mRNA using RNAscope on TA muscle sections from 6-month-old WT and DMD rats. Shown are representative images of tissues from WT (top) and DMD rats (middle) with CDKN2A mRNA appearing as brown dots. Scale bar = 50 μm. A higher magnification image from the dotted area is shown in the bottom frame. Scale bar = 10 μm. The white arrowheads show the mononucleated CDKN2A mRNA^+^ cells. (**e**) Skeletal muscle primary cells from 6-month-old DMD rats were subjected to CDKN2A mRNA in situ hybridisation using RNAscope before the immunocytochemistry of Pax7, CSPG4, CD45, and CD31. Scale bar = 10 μm. White arrowheads indicate DAB signal. White arrows indicate CD45^+^ or CD31^+^ cells. (**f**) Quantification of CDKN2A^+^ Pax7^+^ cells per all Pax7^+^ cells (shown as SC) and CDKN2A^+^ CSPG4^+^ cells per all CSPG4^+^ cells (shown as MPC) in skeletal muscle primary cells from DMD rats (n = 4, each). Data are expressed as mean + SEM, and were compared by Tukey Kramer’s test. For (**a**–**c**), the result of statistical comparison only between the genotypes at each indicated ages was displayed. When a significant age-related difference was observed by the Tukey–Kramer’s test, the † mark was added beside the legend of the graph. *p < 0.05. **p < 0.01. ***p < 0.001.

Senescence is a permanent cell cycle arrest, induced by various genotoxic stressors including oxidative stress^11^. As chronic muscle damage leads to an accumulation of oxidative stress, due to persistent inflammation in DMD^24,30^, we hypothesised that senescence induction in satellite cells as a consequence of oxidative damage may lead to a decrease in their number (Fig. 1k,l) due to cell cycle arrest. To test this hypothesis, we examined the mRNA expression of senescence markers in WT and DMD rat TA muscles. The p16, p19, and p21 expression increased with age in DMD rat skeletal muscle (Fig. 2a–c). To identify the senescent cells in the skeletal muscle, we performed in situ hybridisation of CDKN2A mRNA on TA muscle sections of DMD rats. *CDKN2A* encodes p16 and p19, both of which are well-established senescence markers^13,14^. Therefore, rather than designing a probe specifically targeting p16 mRNAs, we used a probe that covered the region of exon1α and common exon2 and 3, to include p19 mRNA detection. Also, this approach can increase the sensitivity of in situ hybridisation. In situ hybridisation on TA muscle sections of DMD rats showed the expression of CDKN2A mRNA in mononucleated cells (white arrowheads; Fig. 2d). SA-βGal staining is also the well-established method to detect senescent cells. However, this method has a risk of counting macrophages as false positive cells because of their high activity of β-galactosidase^31^ (Supplementary Figure 3a). To minimalize the number of false positive cells, we excluded leukocytes by MACS separation with CD45 antibody in WT and DMD rat primary culture. SA-βGal staining in these cells revealed the increase in the number of SA-βGal^+^ cells in DMD rats compared to WT (Supplementary Figure 3b,c). These results suggest the senescence induction in DMD rat skeletal muscle.

To identify the cell type of senescent cells, we performed in situ hybridisation of CDKN2A mRNA in DMD rat primary cells, followed by immunocytochemistry of various cell type-specific markers. We confirmed the signal of CDKN2A mRNA was not detected in WT rat primary cells (Supplementary Figure 3d). To identify MPCs in the rats, the widely recognised PDGFRα could not be used as a marker due to an unavailability of appropriate antibody for immunocytochemistry. We previously identified chondroitin sulphate proteoglycan 4 (CSPG4) as a specific marker to identify MPCs in rat skeletal muscle^32^. Thus, CSPG4 was employed as a marker for MPCs in the present experiment. Interestingly, the CDKN2A mRNA was expressed not only in Pax7^+^ satellite cells, but also in CSPG4^+^ MPCs (Fig. 2e). Quantitative analysis revealed that, at 6 months, almost 50% of Pax7^+^ cells and 25% of CSPG4^+^ cells expressed CDKN2A (Fig. 2f). These data suggest that senescence was induced both in satellite cells and MPCs.

### Involvement of cellular senescence in the progression of DMD

**Figure 3:**
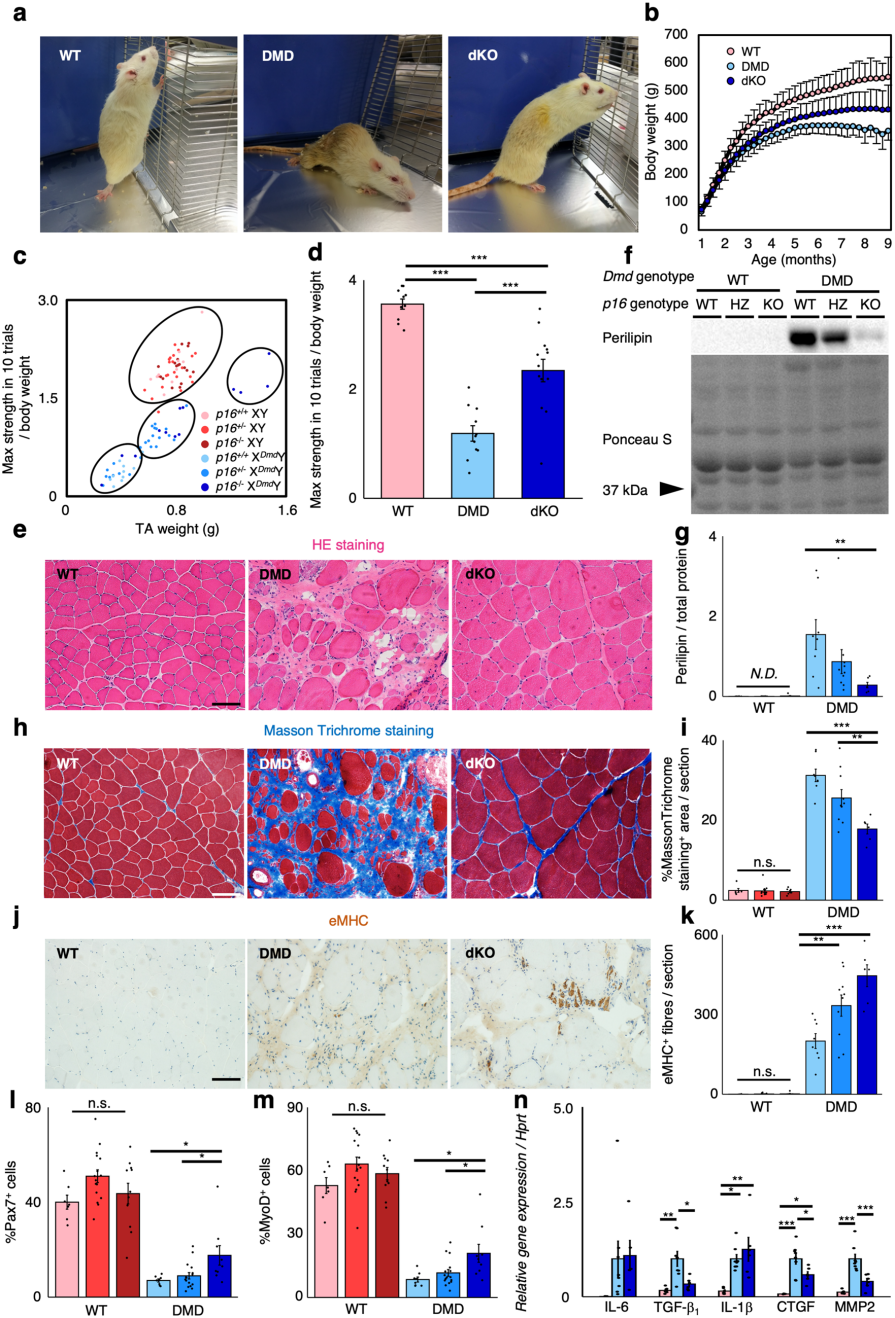
p16KO ameliorates muscular dystrophy in DMD rats. (**a**) Representative images of 9-month-old WT, DMD, and dKO rats. (**b**) Body weight comparison of WT, DMD, and dKO rats of 1–9 months of age (n = 20, 23, 20). (**c**) The plot of maximum muscle strength and TA muscle weight from 9-month-old p16^+/+^, p16^+/−^, and p16^−/−^ background WT and DMD rats (n = 10, 22, 17, 11, 24, 13). Clustering was performed using r mclust package. (**d**) Quantification of maximum muscle strength by grip test in WT, DMD, and dKO rats (n = 10, 11, 13). (**e**) Representative images of HE stains of TA muscles from 9-month-old WT, DMD, and dKO rats. (**f**) Immunoblotting analysis of perilipin expression in 9-month-old p16^+/+^, p16^+/−^, and p16^−/−^ background WT and DMD rats. Full-length blots are presented in Supplementary Figure 6a. (**g**) Quantification of perilipin protein expression (n = 6, 13, 8, 8, 10, 6). (**h**) Masson Trichrome stains of TA muscles from 9-month-old WT, DMD, and dKO rats. (**i**) Quantification of Masson Trichrome staining positive area per section in 9-month-old p16^+/+^, p16^+/−^, and p16^−/−^ background WT and DMD rats (n = 6, 13, 8, 8, 10, 6). (**j**) Immunohistochemical analysis of eMHC in TA muscle sections from 9-month-old WT, DMD, and dKO rats. (**k**) Quantification of eMHC positive fibres per section in 9-month-old p16^+/+^, p16^+/−^, and p16^−/−^ background WT and DMD rats (n = 6, 13, 8, 8, 10, 6). (**l**,**m**) Quantification of (**l**) Pax7^+^ and (**m**) MyoD^+^ cells of skeletal muscle primary cells from 9-month-old p16^+/+^, p16^+/−^, and p16^−/−^ background WT and DMD rats (n = 6, 13, 8, 8, 10, 6). (**n**) Quantification of mRNA levels of SASP markers (IL-6, TGF-β_1_, IL-1β, CTGF, and MMP2) in WT, DMD, and dKO rats (n = 6, 8, 6). Data are expressed as mean ± SEM except for (**b**), which is expressed as mean ± SD, and were compared by Tukey Kramer’s test. For (**g**), (**i**), (**k**–**m**), Tukey Kramer’s test was performed only between WT background groups, or between DMD background groups. For (**g**), (**i**), (**k**–**n**), the colour of each bar indicates the genotype as shown in (**c**). *p < 0.05. **p < 0.01. ***p < 0.001. *n.s. *not significant; *N.D. *not detected; scale bar = 100 μm.

Recent studies have shown the involvement of senescence in the progression of various diseases, such as idiopathic pulmonary disease^19^, hepatic steatosis^20^, atherosclerosis^21^, and others^22,23^. Senescent cells not only stop the cell cycle, but also secrete many cytokines^33,34^. We previously demonstrated that senescent MPCs in aged skeletal muscle disrupt muscle regeneration through SASP^18^. Thus, the presence of senescent MPCs in DMD rat skeletal muscle would have an inhibitory role in muscle regeneration. In addition, the presence of CDKN2A positive satellite cells may have a similar deleterious effect on muscle regeneration, possibly through cell cycle arrest. Therefore, we hypothesised that reversing senescence by genetic ablation of *p16* may lead to recovery of functional satellite cells and MPCs, and a subsequent improvement in the DMD phenotype. We generated *p16* knock-out rats with a CRISPR/Cas system, and crossed them with DMD rats (X^*Dmd*^Y rats) (Supplementary Figure 4a,b). Western blot analysis showed the expression of p16 protein in p16^+/+^ X^*Dmd*^Y rats (DMD rats) while no band was detectable in p16^+/+^ XY rats (WT rats), which is consistent with the result of the elevated levels of CDKN2A mRNA expression in DMD rats in Fig. 2a,b (Supplementary Figure 4c). The complete absence of p16 expression was confirmed in p16^−/−^ X^*Dmd*^Y rats. Approximately half the amount of p16 compared to p16^+/+^ X^*Dmd*^Y rats was detected in p16^+/−^ X^*Dmd*^Y rats, whereas none of p16^+/+^, p16^+/−^, and p16^−/−^ with WT background rats expressed p16 (Supplementary Figure 4c). The p16^−/−^ X^*Dmd*^Y rats (*p16* and *Dmd* double knock-out rats; dKO rats) showed a milder phenotype than DMD rats in fore- and hindlimb paralysis and kyphosis (Fig. 3a). Compared to DMD rats, increased body mass was evident in dKO rats, though it was not to the same level as WT rats (Fig. 3b). The cluster analysis in the plot of TA weight and muscle strength from p16^+/+^, p16^+/−^, and p16^−/−^ WT rats, and p16^+/+^, p16^+/−^, and p16^−/−^ DMD rats revealed there were 4 groups, within which almost all rats with WT background were accumulated in 1 group, and rats with DMD background were divided into 3 groups (Fig. 3c). Almost all DMD rats were in a group that exhibits lower muscle strength and TA weight. However, dKO rats were mainly located in a group that showed lower muscle strength but comparable TA weights to WT rats. Also, some of the dKO rats showed muscle strength similar to WT rats, and their TA weights were even higher than WT rats. Overall, dKO rats showed greater muscle strength than DMD rats, but it was not completely to the same level as WT rats (Fig. 3d). Although no significant difference was observed in serum CK activity in DMD background groups (Supplementary Figure 4d), we observed dramatic improvement of skeletal muscle histology (HE staining) of TA muscle sections from dKO rats, as indicated by a decrease in adipogenesis and fibrosis compared to DMD rats (Fig. 3e). These were confirmed by the observations of immunoblot analysis of perilipin (Fig. 3f,g; Supplementary Figure 6a), and quantification of the Masson Trichrome stained area in TA muscle sections (Fig. 3h,i). These results suggest the role of senescent cells in the progression of DMD.

Further histological analysis revealed an increase in the number of eMHC positive fibres per section in dKO rats (Fig. 3j,k). The *p16* ablation increased the proportion of Pax7 and MyoD positive cells in dKO rats (Fig. 3l,m), suggesting the recovery of satellite cells from cell cycle arrest, and improved muscle regeneration. Moreover, *p16* ablation in DMD rats decreased the expression of some SASP factors, including TGF-β_1_, Connective tissue growth factor (CTGF), and Matrix metalloproteinase 2 (MMP2) (Fig. 3n).

Although *p16* ablation contributes to the amelioration of the dystrophic phenotype, some of the dKO rats developed rhabdomyosarcoma at over 9 months of age (data not shown). Therefore, a novel approach other than inhibition of p16 expression is required for the cure of DMD.

### Senolytic drug ABT263 inhibits the progression of DMD

**Figure 4:**
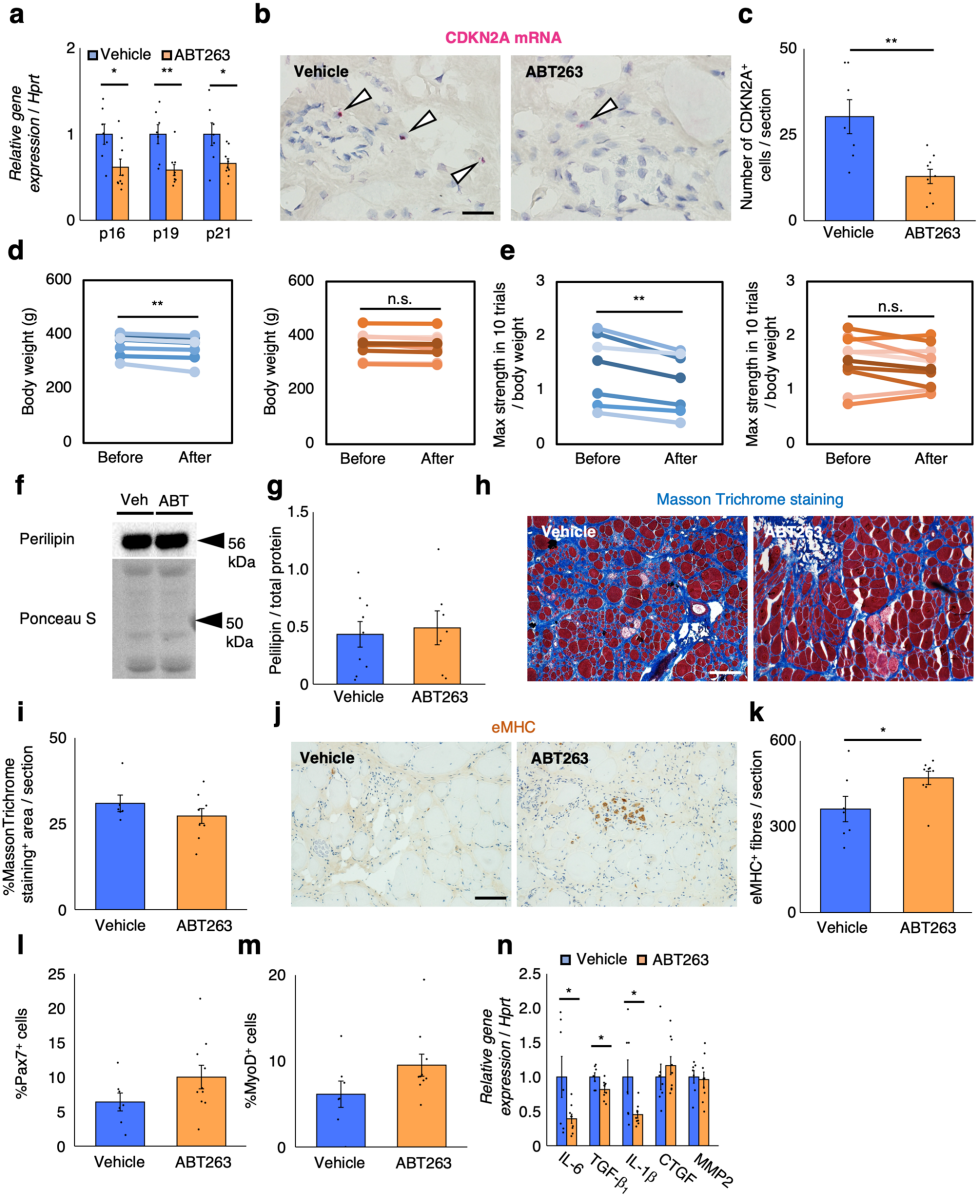
Senolytic drug ABT263 inhibits the exacerbation of muscular dystrophy in DMD rats. (**a**) Quantification of mRNA levels of senescence markers (p16, p19, and p21) in vehicle- and ABT263-treated rats (n = 7, 9). (**b**) In situ hybridisation of CDKN2A mRNA using RNAscope on TA muscle sections from vehicle- or ABT263-treated rats, with CDKN2A mRNA appearing as red dots. The white arrowheads show the CDKN2A mRNA^+^ cells. Scale bar = 50 μm. (**c**) Quantification of the number of CDKN2A mRNA positive mononucleated cells per section from vehicle- or ABT263-treated rats. (**d**) Body weight comparison before and after treatment with vehicle (left panel) or ABT263 (right panel) (n = 7, 9). (**e**) Quantification of maximum muscle strength by grip test before and after treatment with vehicle (left panel) or ABT263 (right panel) (n = 7, 9). (**f**) Immunoblotting analysis of perilipin expression in vehicle- and ABT263-treated rats. Full-length blots are presented in Supplementary Figure 6a. (**g**) Quantification of perilipin protein expression (n = 7, 9). (**h**) Masson Trichrome stains of TA muscles from vehicle and ABT263-treated rats. Scale bar = 100 μm. (**i**) Quantification of Masson Trichrome staining positive area per section (n = 7, 9). (**j**) Immunohistochemical analysis of eMHC in TA muscle sections from vehicle- and ABT263-treated rats. Scale bar = 100 μm. (**k**) Quantification of eMHC positive fibres per section (n = 7, 9). (**l**,**m**) Quantification of (**l**) Pax7^+^ cells and (**m**) MyoD^+^ cells of skeletal muscle primary cells from vehicle- and ABT263-treated rats (n = 7, 9). (**n**) Quantification of mRNA levels of SASP markers (IL-6, TGF-β_1_, IL-1β, CTGF, and MMP2) in vehicle- and ABT263-treated rats (n = 7, 9). Data are expressed as mean ± SEM. The p-value was determined by paired Student’s *t* test for (**d**) and (**e**), and unpaired Student’s *t* test for others. *p < 0.05, **p < 0.01. *n.s. *not significant.

Currently, there are no effective drugs to ameliorate DMD, especially at the late stage of the disease. Based on our above observation that senescent cells appeared in the skeletal muscle of DMD rats, we focused on the senolytic drug ABT263, which induces the specific depletion of senescent cells^35^. Senescent cell removal may not reverse cell cycle arrest in satellite cells. However, since SASP of senescent MPCs inhibits muscle regeneration^18^, decreasing or depleting senescent cells may lead to inhibition of SASP, resulting in increased muscle regeneration. To test this hypothesis, we orally administrated vehicle or ABT263 to 8-month-old DMD rats; this age represented the late stage of DMD. ABT263 was administered to rats p.o. at 18.75 mg/kg body weight/day for 7 days per cycle, for two cycles with a 2-week interval between them. ABT263 treatment successfully decreased the expression of senescence markers p16, p19, and p21 (Fig. 4a). In situ hybridisation of CDKN2A mRNA on TA muscle sections from vehicle- and ABT263-treated rats showed that ABT263 treatment decreased the number of CDKN2A^+^ mononucleated cells (Fig. 4b,c). The vehicle-treated group showed signs of disease progression, such as significant loss of body weight and decline in muscle strength (Fig. 4d,e). While the ABT263-treated group did not show body weight loss or decrease in muscle strength (Fig. 4d,e), the histological analysis showed no significant difference in adipogenesis (Fig. 4f,g; Supplementary Figure 6a) or fibrosis (Fig. 4h,i) between vehicle- and ABT263-treated groups. Immunohistochemical analysis revealed an increase in eMHC positive myofibres (Fig. 4j,k), without an increase in the number of Pax7^+^ or MyoD^+^ cells (Fig. 4l,m). Additionally, quantification of muscle fibre size showed slight increase of them in ABT treated group (Supplementary Figure 5). These results support our notion that the presence of senescent cells is deleterious and affects the disease progression of DMD. Although administration of ABT263 did not increase the number of satellite cells, ABT263 might inhibit the progression of DMD through improving muscle regeneration, even at a late stage of the disease. ABT263 administration decreased the expression of some SASP factors (Fig. 4n). However, in contrast to *p16* ablation, the decrease was only observed in IL-6, TGF-β_1_, and IL-1β expression, while the levels of CTGF and MMP2 were unchanged.

### Senescence induction in DMD patients

**Figure 5:**
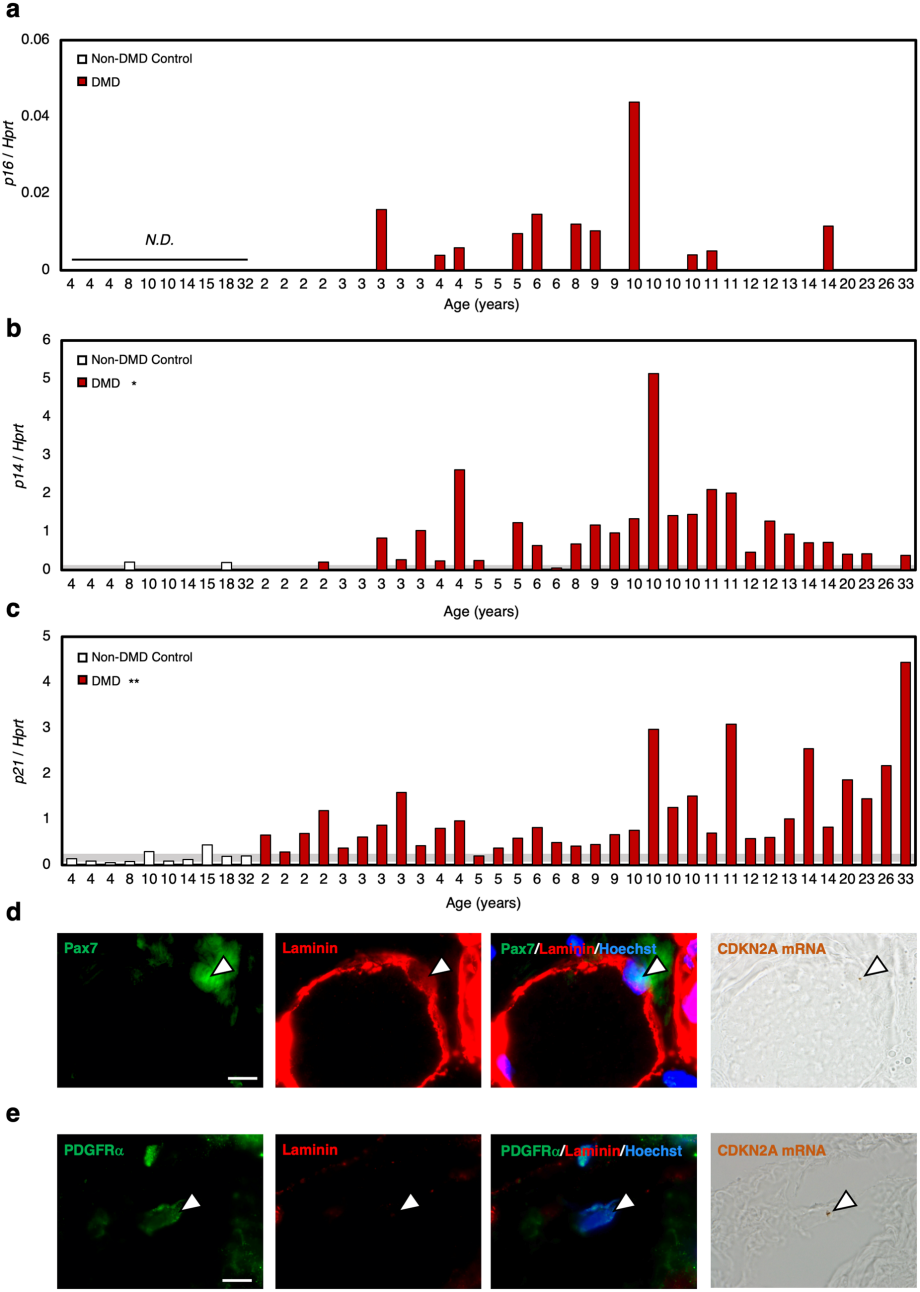
Senescence markers were elevated in human DMD patients. (**a**–**c**) Quantification of mRNA levels of senescence markers (p16, p14, and p21) in non-DMD control individuals and patients with DMD. Individual data from non-DMD control individuals and DMD patients are expressed as bar graphs (n = 10, 35). The grey band behind the graph indicates the mean ± SEM value range of the non-DMD control group. The figure without a grey band indicate that the target gene expression was not observed in the non-DMD control group. (**d**,**e**) Skeletal muscle sections from DMD patients were subjected to in situ hybridisation of CDKN2A mRNA using RNAscope before immunocytochemical analysis of (**d**) Pax7 or (**e**) PDGFRα with laminin. CDKN2A mRNA appears as brown dots. Scale bar = 10 μm. White arrowheads indicate DAB signal. *N.D. *not detected.

To investigate whether the appearance of senescent cells is also observed in patients with DMD, we conducted qPCR analysis of senescence markers in skeletal muscle of non-DMD control individuals and patients with DMD. Thirty-four patients with DMD, between the ages of 2 and 33, and 10 non-DMD control participated in this study. The expression of p16 was detected only in patients with DMD from 3 years and older, and no expression was detected in non-DMD control individuals (Fig. 5a). As for p14 and p21, significantly higher expression was observed in patients with DMD than in non-DMD control individuals. Also, elevated expression of p14 and p21 were observed from as early as 2 years old (Fig. 5b,c). Altogether, these findings suggest that the appearance of senescent cells also occurs in human patients with DMD, and senescence was induced from young age. To identify the cell type of senescent mononucleated cells, we performed in situ hybridisation of CDKN2A mRNA on muscle sections from randomly selected 5 patients with DMD (Patient Number: 12, 25, 30, 35 and 40) and 3 non-DMD controls (Patient Number: 7, 8 and 9). The existence of CDKN2A^+^ Pax7^+^ cells and CDKN2A^+^ PDGFRα^+^ cells was observed in all of the sections from patients with DMD, while not in sections from non-DMD controls. The representative images of CDKN2A^+^ Pax7^+^ cells and CDKN2A^+^ PDGFRα^+^ cells were expressed in Fig. 5d,e. These results suggest the induction of senescence in satellite cells and MPCs in human patients with DMD.

## Discussion

Senescence can be induced by several cell damaging stimuli, such as oncogenic stress, DNA damage stress, and oxidative stress^36^. In DMD, the lack of dystrophin causes the degeneration of skeletal muscle, and sustained leakage of cell cytoplasm into the extracellular milieu triggers innate immune responses, including the binding of damage-associated molecular pattern (DAMP) molecules to Toll like receptors (TLRs) on innate immune cells^37^. This promotes the generation of reactive oxygen species in inflammatory cells, and thus patients with DMD show a progressive increase in the level of oxidative stress in their skeletal muscle^24^. In patients with DMD, inflammatory cell infiltration is evident from at least 2 years old, and prolonged inflammation is observed until the late stages of the disease^27,38^. Another study using DMD rats reported on inflammatory cell infiltration from 2 weeks, which peaked at 8–12 weeks in their skeletal muscle^39^. In mdx mice, the peak inflammation was observed as early as 2–3 weeks of age, and this inflammation subsequently decreased by 12 weeks of age^40^, suggesting inflammatory cell infiltration in skeletal muscles is more continuous in DMD rats than mdx mice. Senescence can also be induced by several cytokines, including TNF-α and TGF-β^41,42^. Elevated expression of these cytokines has been detected in the skeletal muscle of patients with DMD^43,44^, and inflammatory cells are considered the main source of these factors^45,46^. Altogether, continuous inflammatory cell infiltration observed in DMD rats and patients with DMD may cause oxidative stress and senescence-inducing cytokine secretion, ultimately leading to senescence in satellite cells and MPCs. The current results suggest the role of senescence in the exacerbation of DMD. As senescence was induced in satellite cells and MPCs, the involvement of these cells in the progression of DMD was suspected.

Regarding the satellite cells, the impaired proliferation and differentiation might be the cause of exacerbation of DMD. Myoblasts of patients with DMD show decreased myogenic potential^47^. Since absence of dystrophin in satellite cells are reported to lose polarity with impaired asymmetric division, leading to reduction in myogenic progenitors in mdx mice, absence of dystrophin in DMD rats may also affect myogenic commitment of satellite cell in cell intrinsic way^48^. Considering that decrease in the number of MyoD^+^ cells was observed in DMD rats from 1 month of age while there was no significant difference in the number of Pax7^+^ cells between WT and DMD rats at 1 month of age, it is suggested that the impaired myogenic commitment of satellite cells already apparent before the senescence induction. In addition to this cell intrinsic impairment, senescence induction might cause the exacerbation of DMD. A previous report showed the artificial ablation of telomerase in mdx mice led to a more severe phenotype than in normal mdx mice, caused by impaired proliferation and a progressive reduction in the number of satellite cells with age^49^. There is also another report showing the slight decrease in telomere length in mdx mice diaphragms, in which relatively severe phenotype is observed, compared to TA muscles^50^. Since telomere shortening is one of the causes of senescence, these data might represent the effect of senescence in satellite cells in the dystrophic muscle milieu. Senescence in satellite cells in aged muscle causes a decrease in self-renewal, leading to a depletion of the skeletal muscle satellite cell pool and subsequent impaired regeneration^23^. Furthermore, satellite cells in aged mouse skeletal muscle show a decreased proportion of MyoD^+^ cells after muscle injury, indicating less activation of satellite cells^23^. Moreover, DNA damage signalling-mediated senescence in myogenic cells inhibits the myogenic program^51^. Overall, the age-associated decrease in the number of satellite cells and defects in muscle regeneration observed in DMD rats and patients with DMD might be attributed to the impaired proliferation and differentiation of satellite cells.

As for MPCs, secreted factors might be involved in the exacerbation of DMD. A recent study has shown that MPCs contribute to satellite cell pool maintenance. Long term in vivo depletion of MPCs using a PDGFRα-Cre system decreased the number of satellite cells, suggesting a role of MPCs in satellite cell pool maintenance^52^. Our previous research suggested that the SASP of senescent MPCs abrogates myoblast fusion^18^. Recently, another group showed the decreased secretion of Wisp-1 in MPCs from aged mice mediates the age-related decline in muscle regeneration through the inhibition of self-renewal and myogenic commitment of satellite cells^53^. Moreover, MPCs isolated from fibrotic dystrophic muscle show higher secretion of MMP14 and Bone morphogenetic protein1 (BMP1), which exert their effects as activators of TGF-β^54^. Active TGF-β is not only a crucial mediator of fibrogenesis^55^, but also works as an inhibitor of satellite cell proliferation, activation, and differentiation^56,57^. Taken together, in DMD, senescence induction in MPCs may lead to altered cytokine expression, which inhibits satellite cell pool maintenance and/or the myogenic differentiation program. In the present study, ABT263 administration improved the muscle regeneration capacity without an increase in the number of satellite cells. Considering the expression of senescence markers was decreased by ABT263 administration, apoptosis was induced in senescent satellite cells and MPCs. This should decrease the total number of satellite cells, while the number of satellite cells was not changed between the treatments. From the mechanism mentioned above, elimination of senescent MPCs might canceled the inhibitory effect of proliferation on satellite cells, which might compensate the decrease of the satellite cell number. Furthermore, improved muscle regeneration capacity by the ABT263 administration might also be attributed to the killing of senescent MPCs, which might reduce the deleterious effect of SASP on myogenic commitment.

In our research, we identified that both *p16* ablation and ABT263 administration decreased the expression of some SASP factors in the skeletal muscle. However, the change in expression pattern is different between the treatments. Previous studies have shown that senolytic drug treatment successfully decreased the expression of SASP factors in vivo^19,58–60^. In contrast, the effect of *p16* ablation on the expression of SASP factors is rather controversial. Some reports have shown that *p16* ablation decreased the expression of SASP factors and renders the amelioration of the intervertebral disc degeneration and renal tubulointerstitial injury^61,62^. However, other report has shown that genetic inactivation of *p16* does not affect the SASP phenotype or the progression of the disease^63^. Additionally, a previous report showed that cells induced to senesce by the overexpression of p16 do not acquire SASP despite the other hallmarks of senescence^64^, suggesting that p16 is not the direct regulator of SASP, and its role in the inhibition of SASP is context dependent. In our experiments, although ABT263 treatment decreased the expression of IL-6, TGF-β_1_, and IL-1β, it did not change the expression of CTGF and MMP2, which *p16* ablation did. Since mesenchymal fibroblasts secrete both CTGF and MMP2^65,66^, we cannot exclude the possibility that *p16* ablation does not affect the SASP and the decreased levels of these two cytokines is attributed to the ameliorated fibrosis in dKO rats. Further investigation to clarify the effect of *p16* ablation on SASP is required.

Our results demonstrated that successful depletion of mononucleated senescent cells by ABT263 administration increased the muscle regeneration capacity, and thus inhibited the progression of DMD, suggesting the potential application of ABT263 as the therapeutic drug for DMD. Currently, there is no definitive treatment for DMD; while steroids are standardly used to treat DMD, they have severe immune modulatory side effects. Recently, exon skipping and viral vector-based approaches have become the most advanced form of treatments for DMD. However, these treatments have limited efficacy, as well as the potential to induce adverse immune responses^67,68^. Moreover, exon skipping is only applicable to certain patients because of its dependency on the mutation pattern in *DMD*. In our experiments, we discovered that senescence might be a novel treatment target for DMD. Inhibiting the expression of p16 ameliorated the severity of DMD, but increased the risk of carcinogenesis. Senolytic drug treatment inhibited the progression of DMD even in the late stages of the disease. As senolytic drugs do not depend on immune-modulation or genetic background, these may overcome the disadvantages of steroid and exon skipping treatments. In addition, senolytic drugs do not need to be continuously present in the systemic circulation. For example, the single administration of a combination drug of dasatinib and quercetin, other senolytic treatment, every two weeks extends both the health- and life-span in aged mice^59^, indicating that senescence development might take more than 2 weeks in aged mice. In our experiments, the expression of senescence markers was very low in the DMD rats at 1 month of age, comparable level to that in the age-matched WT rats, in spite of the chronic inflammation. Another group using DMD rats revealed inflammatory cell infiltration from 2 weeks^39^; hence, senescent cell development may take more than 2 weeks in DMD rats. These results suggest that intermittent administration might be effective to treat DMD which reduces the risk of side-effects. Taken together, senolytic drugs show potential application in DMD treatment, which will elicit fewer adverse side-effects and benefit more patients than alternative genetic approaches. However, the present study has a limitation that there are no experiments of ABT263 administration to WT rats. Previous studies have demonstrated that there are no apparent side effects of ABT263 when treated to WT mice in their brain^69^ or hematopoietic cells^35^. However, the possible side effects should still be further investigated in skeletal muscle. Also, the dosage and time of administration to DMD rats should be further explored before proposing its possible employment in therapeutic protocols in human patients with DMD.

In our research, the expression pattern of senescence markers was different between the rats and humans. Senescence is typically induced by ARF-p53-p21 pathway and/or the p16-pRB pathway. However, the pathways by which cells become senescent are cell-type specific, and species specific^70^. Considering DMD rats have high levels of p16 while patients with DMD exhibit high expression of p14^ARF^ and p21, senescence induction pathway is different between the species in skeletal muscle. However, further studies are needed to investigate the mechanism of senescence induction both in rats and humans.

In conclusion, our data demonstrate that senescence mediates the exacerbation of DMD. As our data suggests the induction of senescence in patients with DMD, targeting senescent cells may serve as a potential novel treatment approach. Other forms of muscular dystrophies, such as Merosin deficient congenital muscular dystrophy type 1A (MDC1A) or BMD, also manifest chronic muscle fibre degeneration and inflammation^71,72^, from which we can assume that senescence plays a role. Our study offers new insight into the integral role of senescent cells in the progression of DMD and provides a basis for the development of novel therapies for patients with DMD and other muscular dystrophies.

## Materials and methods

### Patients and donors

Biopsy or autopsy samples of patients with DMD were used for the experiments, courtesy of Drs. Nishino and Ogasawara from the National Center of Neurology and Psychiatry. These trials were approved by the University-wide Ethics Review Committee of the University of Tokyo (19-322) and the Ethics Committee of the National Center of Neurology and Psychiatry (A2019-031) and have been performed in accordance with the ethical standard laid down in an appropriate version of the 2013 Declaration of Helsinki. The experiments were conducted with the informed consent of the patients, and their parents, where applicable. Thirty-four patients with DMD, between the ages of 2 and 33, participated in this study. Dystrophin gene mutations were identified in 23 patients, and the remaining patients were diagnosed as DMD without genetic testing, through exhibiting typical clinical signs and sections of their biopsy or autopsy samples had a lower immunoreactivity to dystrophin protein (Supplementary Table). Non-DMD control biopsies were from individuals in whom neuromuscular disease was suspected, but did not show any sign of histological pathology on their skeletal muscle sections.

### Animals

We chose to use the previously established^26^ DMD rat model, carrying mutations in exon3 and exon16 (Supplementary Figure 1a) in *Dmd*, because it has a more severe disease phenotype and is more representative of the late-stage DMD in humans than the broadly used mouse model. X^*Dmd*^X female rats were mated with wild-type (WT) adult male rats to generate male WT rats and DMD rats, which were used for experiments. Animals were maintained under controlled environmental conditions, at 23 °C, with a light/dark (12/12 h) cycle (lights on at 0800), and food and water were provided ad libitum. All animal experiments performed in this study were in accordance with the Guide for the Care and Use of Laboratory Animals of the University of Tokyo, and were approved (P18-125) by the Institutional Animal Care and Use Committee of the University of Tokyo.

### Generation of p16KO rats with the CRISPR/Cas system

The choice of target sites and the construction of Cas9 mRNA or gRNAs were performed according to previously published methods^26^. Five-week-old female Wistar-Imamichi rats were superovulated by intraperitoneal injection of 25 IU equine chorionic gonadotropin (eCG) followed by 25 IU human chorionic gonadotropin (hCG) at intervals of 48 h and mated with male WT rats. Around 20 h post-hCG injection, zygotes were collected, and approximately 4 pL of a mixture of 10 μg/mL gRNAs and 10 μg/mL Cas9 mRNA was injected into each zygote using a micro-injector (Narishige, Tokyo, Japan). After 2 h incubation in M16 medium, zygotes were transferred into the oviductal ampullas of 8-week-old pseudopregnant rats. After birth, 1–2-mm tips of the tails were obtained from the new-born rats and used for genomic DNA extraction. PCR was performed to confirm the deletion in the target gene. The primers used for PCR were as follows; forward, 5′-CAC TGA ATC TCC GAG AGG AAG G-3′; reverse, 5′-ATT ACC TGG GGT ATA CAT TTC ATG C-3′. PCR products were purified by agarose gel electrophoresis and subsequently sequenced as previously reported^26^. To exclude the effect of off-target, we mated the F0 p16^−/−^ rats with WT rats further than F6. The male p16^−/−^ X^*Dmd*^Y rats were generated by crossing p16^+/−^ X^*Dmd*^X rats with p16^+/−^ XY rats. Male littermates, p16^+/+^ XY, p16^+/−^ XY, p16^−/−^ XY, p16^+/+^ X^*Dmd*^Y, and p16^+/−^ X^*Dmd*^Y, were used as controls.

### Grip test

Forelimb strength (kg) was determined using a grip strength metre (Melquest, Toyama, Japan). Tails of rats gripping a T-shaped bar, which was connected to a monitoring devise, were pulled horizontally and the maximum value was recorded. In 10 serial times of pulling, the maximum forelimb muscle strength was used as the value for muscle strength.

### Histological analyses

Frozen sections (7–8 μm thick) of Tibialis anterior (TA) muscles and paraffin-embedded sections of diaphragm were prepared transversely and subjected to histological analyses. The sections were used for haematoxylin and eosin (HE), Masson’s trichrome staining, and immunostaining.

For immunostaining, cryosections were fixed with 4% paraformaldehyde, blocked with 5% normal goat serum in phosphate-buffered saline (PBS), and incubated overnight at 4 °C with anti-eMHC primary antibodies (1:100, mouse, clone F1.652; Developmental Studies Hybridoma Bank, Iowa City, IA, USA). The following day, the sections were washed and incubated for 1 h with Histofine Simple stain Rat MAX-PO (Nichirei Biosciences Inc., Tokyo, Japan). After the DAB reaction, nuclei were counterstained with haematoxylin. Photographs were acquired using a fluorescence microscope (BX51, Olympus, Tokyo, Japan) equipped with a digital camera (DP73, Olympus).

For the immunohistochemical analysis of human muscle tissues, biopsied or autopsied samples were cryosectioned, fixed with 4% paraformaldehyde, blocked with 5% normal donkey serum in PBS, and incubated overnight at 4 °C with anti-Pax7 (1:100, mouse, clone P3U1; Developmental Studies Hybridoma Bank) or anti-PDGFRα (2.5 μg/mL; AF-307-NA; R&D, Minneapolis, MN, USA) along with anti-laminin (1:100, rabbit, Sigma, St Louis, MO, USA) primary antibodies. The following day, samples were incubated with AlexaFluor-conjugated secondary antibodies (1:500; Jackson Immunoresearch Laboratories, West Grove, PA, USA) for 1 h, and nuclei were counterstained with Hoechst 33258.

For the quantification of myofibre size, we used CellProfiler (version 3.1.8) and Fiji (version 2.0.0-rc-69/1.52p). Briefly, from the image of Masson’s trichrome staining, we extracted the area of red stained myofibres using CellProfiler. Each myofibres were identified as particles in Fiji, and the minimum feret diameter was calculated.

### Isolation of rat skeletal muscle primary cells

Protocols for isolating primary cells from skeletal muscles were described previously^73^. Briefly, rats were euthanised by CO_2_ inhalation, and their quadriceps femoris muscles were subsequently separated from the associated fat and connective tissue. After isolation, they were hand-minced, and digested for 1 h at 37 °C with 1.25 mg/mL protease (from *Streptomyces griseus*, type XIV; Sigma). Cells were separated from myofibre fragments through differential centrifugation and plated on both poly-l-lysine- and fibronectin-coated plates. Cells were cultured in Dulbecco’s modified Eagle medium (Gibco, Life Technologies, Palo Alto, CA, USA) containing 10% foetal bovine serum, 100 U/mL penicillin, 100 mg/mL streptomycin, and 50 mg/mL gentamicin for 2 days before use in the experiments.

### Immunocytochemistry

For immunostaining, cells were fixed with 4% paraformaldehyde, blocked with 5% normal goat serum in PBS containing 0.1% Triton X-100 (Sigma), and incubated overnight with primary antibodies (detailed below) at 4 °C. The following day, they were incubated with AlexaFluor-conjugated secondary antibodies (1:500; Invitrogen, Carlsbad, CA, USA) for 1 h, and nuclei were counterstained with Hoechst 33258. The Pax7^+^ and MyoD^+^ cells were counted in 5 randomly selected fields at 10 × the objective of a fluorescence microscope (BX50, Olympus). Photographs were acquired using the BX50 fluorescence microscope equipped with a digital camera (DP70, Olympus). Mean pixel measurements were obtained using ImageJ software (v1.47; National Institutes of Health, Bethesda, MD, USA). The number of Pax7^+^ or MyoD^+^ cells was counted using an ImageJ plugin provided by Computer Vision Group in University of Freiburg, which enabled deep learning-based analysis with U-net^74^. AWS amazon cloud p2.xlarge instance was used as a remote cloud GPU computer. Several images were annotated manually as training data, and used for finetuning. After optimisation, all images were analysed by the finetuned model.

The following primary antibodies were used: anti-Pax7 (1:200, mouse, clone P3U1; Developmental Studies Hybridoma Bank), anti-MyoD (1:200, mouse, clone 5.8A; Novocastra, Newcastle upon Tyne, UK), anti-CSPG4 [1:50, mouse, clone 5C12, produced in our laboratory^32^], anti-CD45 (1:100, mouse, clone OX-1; BD Biosciences, Franklin Lakes, NJ, USA), and anti-CD31 (1:100, rabbit, RB-10333-P0; Funakoshi, Tokyo, Japan).

### Immunoblotting

Frozen TA muscle sections were lysed in RIPA buffer (50 mM Tris–HCl pH 7.4, 1% NP-40, 0.5% Na-deoxycholate, 0.1% sodium dodecyl sulphate (SDS), 150 mM NaCl, 2 mM EDTA, and 50 mM NaF), and the protein extracts were subsequently separated on SDS–polyacrylamide gels. After the electrophoresis, proteins were electroblotted onto polyvinylidene fluoride membranes, followed by staining with Ponceau S staining solution (0.1% (w/v) Ponceau S in 5% (v/v) acetic acid). Then, membranes were blocked with 5% skimmed milk/PBS for 1 h. Perilipin, p16, and dystrophin protein were detected by staining with anti-perilipin antibody (1:1000, rabbit, #3470; Cell Signaling Technology, Danvers, MA, USA), anti-p16 antibody (1:4000, rabbit, ab211542; Abcam, Cambridge, UK), and anti-dystrophin antibody (1:400, rabbit, ab154168, detecting aa3650 to C-terminus; Abcam), respectively, followed by incubation with anti-rabbit IgG horseradish peroxidase-labelled second antibody (1:8000, goat, 111–035-144; Jackson ImmunoResearch Laboratory). Bands were visualised using an ECL western blotting analysis system (GE Healthcare Life Science, Buckinghamshire, UK).

### Reverse transcription-PCR (RT-PCR)

Protocols for RNA extraction or quantitative RT-PCR were described previously^18^. Briefly, total RNA was extracted from rat frozen TA muscle sections with TRIzol Reagent (Invitrogen) and human cryosectioned biopsy samples, and cDNA was synthesised using a Super Script II kit (Invitrogen). Quantitative RT-PCR (qPCR) was performed on a Light Cycler 2.0 (Roche Diagnostics, Roche, Basel, Switzerland), using a Thunderbird SYBR qPCR Mix (TOYOBO, Osaka, Japan). For qPCR, the following primer sets were used for the rat samples (with an annealing temperature of 60 °C in all cases): p16: forward, 5′-TTC ACC AAA CGC CCC GAA CA-3′; reverse, 5′-CAG GAG AGC TGC CAC TTT GAC-3′; p19: forward, 5′-GTG TTG AGG CCA GAG AGG AT-3′; reverse, 5′-TTG CCC ATC ATC ATC ACC T-3′; p21: forward, 5′-GAC ATC TCA GGG CCG AAA-3′; reverse, 5′-GGC GCT TGG AGT GAT AGA AA-3′; IL-6: forward, 5′-CCT GGA GTT TGT GAA GAA CAA CT-3′; reverse, 5′-GGA AGT TGG GGT AGG AAG GA-3′; TGF-β1: forward, 5′-CCT GGA AAG GGC TCA ACA C-3′; reverse, 5′-CAG TTC TTC TCT GTG GAG CTG A-3′; IL-1β: forward, 5′-TGT GAT GAA AGA CGG CAC AC-3′; reverse, 5′- CTT CTT CTT TGG GTA TTG TTT GG-3′; CTGF: forward, 5′- GGT GAC CTA GAG GAA AAC ATT AAG A-3′; reverse, 5′-CCG GTA GGT CTT CAC ACT GG-3′; MMP2: forward, 5′-CAC CAC CGA GGA TTA TGA CC-3′; reverse, 5′-CAC CCA CAG TGG ACA TAG CA-3′, and hypoxanthine–guanine phosphoribosyltransferase (Hprt): forward, 5′-GAC CGG TTC TGT CAT GTC G-3′; reverse, 5′-ACC TGG TTC ATC ATC ACT AAT CAC-3′. The following primer sets were used for the human samples (with an annealing temperature of 60 °C in all cases): p16: forward, 5′-CTG CCC AAC GCA CCG AAT AG-3′; reverse, 5′-CAC GGG TCG GGT GAG AGT-3′; p14: forward, 5′-CTA CTG AGG AGC CAG CGT CTA-3′; reverse, 5′-CTG CCC ATC ATC ATG ACC T-3′; p21: forward, 5′-CCG AAG TCA GTT CCT TGT GG-3′; reverse, 5′-CAT GGG TTC TGA CGG ACA T-3′, and Hprt: forward, 5′-TGA CCT TGA TTT ATT TTG CAT ACC-3′; reverse, 5′-CGA GCA AGA CGT TCA GTC CT-3′. The expression of each gene was analysed using the crossing-point method.

### In situ hybridisation of CDKN2A mRNA

For in situ hybridization of CDKN2A mRNA on freshly frozen rat TA sections, human cryosectioned biopsy samples, and rat skeletal muscle primary cells, we used the RNAscope Kit (RNAscope 2.5HD Reagent kit Brown or Red; Cosmo Bio Co. Ltd.; Tokyo, Japan). Briefly, muscle sections or cells were fixed with 4% PFA/PBS for 15 min, endogenous peroxidase activity was quenched with RNAscope Hydrogen Peroxide (Cosmo Bio), which was followed by incubation with RNAscope Protease 4 (Cosmo Bio) for 30 min. For cells, cells were incubated with RNAscope Protease 4 for 10 min, followed by incubation with 0.1% Triton/PBS to permeabilise cell membranes. Then, the samples were hybridised with RNAscope Probe-Rn-CDKN2A (for rat samples; Cosmo Bio) or RNAscope Probe-Hs-CDKN2A (for human samples; Cosmo Bio) for 2 h, and signals were amplified with AMP1 to 6 (Cosmo Bio). After the DAB or Fast red reaction, nuclei were counterstained with haematoxylin for rat TA sections while others were counterstained with Hoechst 33258. To identify the cell type of CDKN2A mRNA^+^ cells in skeletal muscle primary cells, we performed immunocytochemistry for Pax7, CSPG4, CD45, and CD31 after the in situ hybridisation.

### SA-βGal staining

We performed SA-βGal staining using kit (Cell Signaling; cat. no. 9860). Briefly, skeletal muscle primary cells were isolated from 3-months-old WT and DMD rats as described above. To exclude the leukocytes including macrophages, we performed MACS separation using CD45 microbeads (Miltenyi Biotec; Bergisch Gladbach, Germany). After elimination of leukocytes, cells were fixed for 10 min with fixation buffer, followed by 15 h incubation in staining solution at 37 °C.

### Administration of ABT263

DMD rats were treated with vehicle (ethanol: polyethylene glycol 400: Phosal 50 PG (standardised phosphatidylcholine (PC) concentrate with at least 50% PC and propylene glycol; Phospholipid Gmbh, Cologne, Germany) at 1:3:6), or ABT263 (in ethanol: polyethylene glycol 400: Phosal 50 PG at 1:3:6). The body weight of the rats was measured daily, and ABT263 was administered to rats p.o. at 18.75 mg/kg body weight/day for 7 days per cycle, for two cycles with a 2-week interval between them.

### Creatine kinase activity

Creatine kinase activity in the sera of DMD rats and their age-matched WT rats (from 3 to 10 months of age) was evaluated using the Fuji Drychem system (Fuji Film Medical Co. Ltd.; Tokyo, Japan).

### Urine titin

Urine samples were obtained from 1- to 11-month-old WT and DMD rats. Urinary titin was measured using a Titin-N Fragment Assay ELISA Kit (Immuno-Biological Laboratories Co. Ltd., Fujioka, Japan), as previously described^75^. Urinary creatinine (Cr) concentrations were measured using an assay kit (LabAssay Creatinine, Wako Pure Chemical Industries, Ltd., Osaka, Japan).

### Measurement of respiratory function

The respiratory function of DMD rats and their age-matched WT rats (from 6 to 10 months of age) was measured with a FinePoint Pulmonary Function Testing (PFT) system (Primetech, Tokyo, Japan). Conscious rats were placed in a box with a whole-body plethysmography system, and the respiratory parameters were recorded for 30 min. Among these data, we chose approximately 30 s when rats were sleeping, to calculate mean Tidal volume breathing (TVb).

### Statistical analyses

The unpaired two-tailed Student's *t* test was used to examine statistical differences between two groups. For the experiment to assess the effect of ABT263 on body weight and muscle strength, the p-value was determined using a paired two-tailed Student's *t* test. For the experiment to assess the effect of ABT263 on myofibre size, the p-value was determined using Wilcoxon rank sum test. Tukey Kramer’s test was used for multiple group comparisons. For Figs. 1c,h,k,l, 2a–c, and Supplementary Figure 1d,e, the result of statistical comparison only between the genotypes at each indicated ages was displayed. When a significant age-related difference was observed by the Tukey–Kramer’s test, the † mark was added beside the legend of the graph. P-values less than 0.05 were considered statistically significant. Data are presented as mean ± SEM except for the data of body weights, which are presented as mean ± SD.

## Supplementary information

Supplementary Information.

## Data Availability

All relevant data are available from authors.

## References

[1] Ryder S (2017). The burden, epidemiology, costs and treatment for Duchenne muscular dystrophy: an evidence review. Orphanet J. Rare Dis..

[2] Mercuri E, Muntoni F (2013). Muscular dystrophies. Lancet.

[3] Yin H, Price F, Rudnicki MA (2013). Satellite cells and the muscle stem cell niche. Physiol. Rev..

[4] Verhaart IEC, Aartsma-Rus A (2019). Therapeutic developments for Duchenne muscular dystrophy. Nat. Rev. Neurol..

[5] Visser M (2002). Relationship of interleukin-6 and tumor necrosis factor-α with muscle mass and muscle strength in elderly men and women: the Health ABC Study. J. Gerontol. Ser. A Biol. Sci. Med. Sci..

[6] Mann CJ (2011). Aberrant repair and fibrosis development in skeletal muscle. Skelet. Muscle.

[7] Joe AWB (2010). Muscle injury activates resident fibro/adipogenic progenitors that facilitate myogenesis. Nat. Cell Biol..

[8] Uezumi A, Fukada S-I, Yamamoto N, Takeda SI, Tsuchida K (2010). Mesenchymal progenitors distinct from satellite cells contribute to ectopic fat cell formation in skeletal muscle. Nat. Cell Biol..

[9] Uezumi A (2011). Fibrosis and adipogenesis originate from a common mesenchymal progenitor in skeletal muscle. J. Cell Sci..

[10] Parrinello S (2003). Oxygen sensitivity severely limits the replicative lifespan of murine fibroblasts. Nat. Cell Biol..

[11] Pole A, Dimri M, Dimri GP (2016). Oxidative stress, cellular senescence and ageing. AIMS Mol. Sci..

[12] Krishnamurthy J (2004). Ink4a/Arf expression is a biomarker of aging. J. Clin. Investig..

[13] Kim WY, Sharpless NE (2006). The regulation of INK4/ARF in cancer and aging. Cell.

[14] Muñoz-Espín D, Serrano M (2014). Cellular senescence: from physiology to pathology. Nat. Rev. Mol. Cell Biol..

[15] Sharpless NE, Chin L (2003). The INK4a/ARF locus and melanoma. Oncogene.

[16] Kuilman T (2008). Oncogene-induced senescence relayed by an interleukin-dependent inflammatory network. Cell.

[17] Tominaga K, Suzuki HI (2019). TGF-β signaling in cellular senescence and aging-related pathology. Int. J. Mol. Sci..

[18] Sugihara H, Teramoto N, Yamanouchi K, Matsuwaki T, Nishihara M (2018). Oxidative stress-mediated senescence in mesenchymal progenitor cells causes the loss of their fibro/adipogenic potential and abrogates myoblast fusion. Aging (Albany NY).

[19] Schafer MJ (2017). Cellular senescence mediates fibrotic pulmonary disease. Nat. Commun..

[20] Ogrodnik M (2017). Cellular senescence drives age-dependent hepatic steatosis. Nat. Commun..

[21] Childs BG (2016). Senescent intimal foam cells are deleterious at all stages of atherosclerosis. Science.

[22] Prattichizzo F (2018). Short-term sustained hyperglycaemia fosters an archetypal senescence-associated secretory phenotype in endothelial cells and macrophages. Redox Biol..

[23] Sousa-Victor P (2014). Geriatric muscle stem cells switch reversible quiescence into senescence. Nature.

[24] Petrillo S (2017). Oxidative stress in Duchenne muscular dystrophy: focus on the NRF2 redox pathway. Hum. Mol. Genet..

[25] Grady RM (1997). Skeletal and cardiac myopathies in mice lacking utrophin and dystrophin: a model for Duchenne muscular dystrophy. Cell.

[26] Nakamura K (2014). Generation of muscular dystrophy model rats with a CRISPR/Cas system. Sci. Rep..

[27] Skuk D (2006). Dystrophin expression in muscles of Duchenne muscular dystrophy patients after high-density injections of normal myogenic cells. J. Neuropathol. Exp. Neurol..

[28] Mauro A (1961). Satellite cell of skeletal muscle fibers. J. Biophys. Biochem. Cytol..

[29] Dumont NA, Bentzinger CF, Sincennes MC, Rudnicki MA (2011). Satellite cells and skeletal muscle regeneration. Compr. Physiol..

[30] Terrill JR (2016). Levels of inflammation and oxidative stress, and a role for taurine in dystropathology of the Golden Retriever Muscular Dystrophy dog model for Duchenne Muscular Dystrophy. Redox Biol..

[31] Brusuker I, Rhodes JM, Goldman R (1982). β-Galactosidase—an indicator of the maturational stage of mouse and human mononuclear phagocytes. J. Cell. Physiol..

[32] Takeuchi S (2016). Roles of chondroitin sulfate proteoglycan 4 in fibrogenic/adipogenic differentiation in skeletal muscle tissues. Exp. Cell Res..

[33] Takahashi A (2012). DNA damage signaling triggers degradation of histone methyltransferases through APC/CCdh1 in senescent cells. Mol. Cell.

[34] Coppé J-P, Desprez P-Y, Krtolica A, Campisi J (2010). The senescence-associated secretory phenotype: the dark side of tumor suppression. Annu. Rev. Pathol..

[35] Chang J (2016). Clearance of senescent cells by ABT263 rejuvenates aged hematopoietic stem cells in mice. Nat. Med..

[36] Hernandez-Segura A, Nehme J, Demaria M (2018). Hallmarks of cellular senescence. Trends Cell Biol..

[37] Rosenberg AS (2015). Immune-mediated pathology in Duchenne muscular dystrophy. Sci. Transl. Med..

[38] Engel AG, Arahata K (1986). Mononuclear cells in myopathies: quantitation of functionally distinct subsets, recognition of antigen-specific cell-mediated cytotoxicity in some diseases, and implications for the pathogenesis of the different inflammatory myopathies. Hum. Pathol..

[39] Ouisse LH (2019). Immunophenotype of a rat model of Duchenne's disease and demonstration of improved muscle strength after anti-CD45RC antibody treatment. Front. Immunol..

[40] Spencer MJ, Walsh CM, Dorshkind KA, Rodriguez EM, Tidball JG (1997). Myonuclear apoptosis in dystrophic mdx muscle occurs by perforin-mediated cytotoxicity. J. Clin. Investig..

[41] Braumüller H (2013). T-helper-1-cell cytokines drive cancer into senescence. Nature.

[42] Reimann M (2010). Tumor stroma-derived TGF-beta limits myc-driven lymphomagenesis via Suv39h1-dependent senescence. Cancer Cell.

[43] Porreca E (1999). Haemostatic abnormalities, cardiac involvement and serum tumor necrosis factor levels in X-linked dystrophic patients. Thromb. Haemost..

[44] Song Y (2017). Expression levels of TGF-β1 and CTGF are associated with the severity of Duchenne muscular dystrophy. Exp. Ther. Med..

[45] De Paepe B, De Bleecker JL (2013). Cytokines and chemokines as regulators of skeletal muscle inflammation: presenting the case of Duchenne muscular dystrophy. Mediat. Inflamm..

[46] Zhou L (2006). Temporal and spatial mRNA expression patterns of TGF-beta1, 2, 3 and TbetaRI, II, III in skeletal muscles of mdx mice. Neuromuscul. Disord..

[47] Delaporte C, Dehaupas M, Fardeau M (1984). Comparison between the growth pattern of cell cultures from normal and Duchenne dystrophy muscle. J. Neurol. Sci..

[48] Dumont NA (2015). Dystrophin expression in muscle stem cells regulates their polarity and asymmetric division. Nat. Med..

[49] Sacco A (2010). Short telomeres and stem cell exhaustion model Duchenne muscular dystrophy in mdx/mTR mice. Cell.

[50] Vita GL (2020). Effect of exercise on telomere length and telomere proteins expression in mdx mice. Mol. Cell. Biochem..

[51] Latella L (2017). DNA damage signaling mediates the functional antagonism between replicative senescence and terminal muscle differentiation. Genes Dev.

[52] Wosczyna MN (2019). Mesenchymal stromal cells are required for regeneration and homeostatic maintenance of skeletal muscle. Cell Rep..

[53] Lukjanenko L (2019). Aging disrupts muscle stem cell function by impairing matricellular WISP1 secretion from fibro-adipogenic progenitors. Cell Stem Cell.

[54] Juban G (2018). AMPK activation regulates LTBP4-dependent TGF-β1 secretion by pro-inflammatory macrophages and controls fibrosis in Duchenne muscular dystrophy. Cell Rep..

[55] Meng X-M, Nikolic-Paterson DJ, Lan HY (2016). TGF-β: the master regulator of fibrosis. Nat. Rev. Nephrol..

[56] Allen RE, Boxhorn LK (1987). Inhibition of skeletal muscle satellite cell differentiation by transforming growth factor-beta. J. Cell. Physiol..

[57] Carlson ME, Hsu M, Conboy IM (2008). Imbalance between pSmad3 and Notch induces CDK inhibitors in old muscle stem cells. Nature.

[58] Zhang P (2019). Senolytic therapy alleviates Aβ-associated oligodendrocyte progenitor cell senescence and cognitive deficits in an Alzheimer's disease model. Nat. Neurosci..

[59] Xu M (2018). Senolytics improve physical function and increase lifespan in old age. Nat. Med..

[60] Ogrodnik M (2019). Obesity-induced cellular senescence drives anxiety and impairs neurogenesis. Cell Metab..

[61] Novais EJ, Diekman BO, Shapiro IM, Risbud MV (2019). p16Ink4a deletion in cells of the intervertebral disc affects their matrix homeostasis and senescence associated secretory phenotype without altering onset of senescence. Matrix Biol..

[62] Jin J (2017). P16 INK4a deletion ameliorated renal tubulointerstitial injury in a stress-induced premature senescence model of Bmi-1 deficiency. Sci. Rep..

[63] Sundar IK, Rashid K, Gerloff J, Li D, Rahman I (2018). Genetic ablation of p16INK4a does not protect against cellular senescence in mouse models of chronic obstructive pulmonary disease/emphysema. Am. J. Respir. Cell Mol. Biol..

[64] Coppé J-P (2011). Tumor suppressor and aging biomarker p16INK4a induces cellular senescence without the associated inflammatory secretory phenotype. J. Biol. Chem..

[65] Sun G (2008). Connective tissue growth factor is overexpressed in muscles of human muscular dystrophy. J. Neurol. Sci..

[66] von Moers A (2005). Increased mRNA expression of tissue inhibitors of metalloproteinase-1 and-2 in Duchenne muscular dystrophy. Acta Neuropathol..

[67] Shimizu-Motohashi Y, Murakami T, Kimura E, Komaki H, Watanabe N (2018). Exon skipping for Duchenne muscular dystrophy: a systematic review and meta-analysis. Orphanet J. Rare Dis..

[68] Motohashi N, Shimizu-Motohashi Y, Roberts TC, Aoki Y (2019). Potential therapies using myogenic stem cells combined with bio-engineering approaches for treatment of muscular dystrophies. Cells.

[69] Bussian TJ (2018). Clearance of senescent glial cells prevents tau-dependent pathology and cognitive decline. Nature.

[70] Itahana K, Campisi J, Dimri GP (2004). Mechanisms of cellular senescence in human and mouse cells. Biogerontology.

[71] Mehuron T (2014). Dysregulation of matricellular proteins is an early signature of pathology in laminin-deficient muscular dystrophy. Skelet. Muscle.

[72] Kaido M, Arahata K, Hoffman EP, Nonaka I, Sugita H (1991). Muscle histology in Becker muscular dystrophy. Muscle Nerve.

[73] Yamanouchi K, Nakamura K, Takegahara Y, Nakano SI, Nishihara M (2013). Ex vivo bupivacaine treatment results in increased adipogenesis of skeletal muscle cells in the rat. Anim. Sci. J..

[74] Falk T (2019). U-Net: deep learning for cell counting, detection, and morphometry. Nat. Methods.

[75] Matsuo M, Awano H, Maruyama N, Nishio H (2019). Titin fragment in urine: a noninvasive biomarker of muscle degradation. Adv. Clin. Chem..

