# Supplementary material for: Cellular senescence-mediated exacerbation of Duchenne muscular dystrophy
Source: Sci Rep. 2020 Oct 12;10:16385. doi: 10.1038/s41598-020-73315-6 (PMC7550355; doi:10.1038/s41598-020-73315-6)
Supplement: Supplementary file 1 — Supplementary Information. [file 41598_2020_73315_MOESM1_ESM.pdf]

Supplementary Files

Title: Cellular senescence-mediated exacerbation of Duchenne muscular dystrophy

Authors

Hidetoshi Sugihara[1], Naomi Teramoto[1], Katsuyuki Nakamura[1], Takanori Shiga[2], Taku Shirakawa[3,4], Masafumi Matsuo[3,4],

Masashi Ogasawara[5,6], Ichizo Nishino[5,6], Takashi Matsuwaki[1], Masugi Nishihara[1], Keitaro Yamanouchi[1,*]

Supplementary Table

Patients	Age	Diagnosis	Mutation	Specimen	Nationality
1	4	non-DMD	No sign of muscle pathology on histology.	Biopsy	Japan
2	4	non-DMD	No sign of muscle pathology on histology.	Biopsy	Japan
3	4	non-DMD	No sign of muscle pathology on histology.	Biopsy	Japan
4	8	non-DMD	No sign of muscle pathology on histology.	Biopsy	Japan
5	10	non-DMD	No sign of muscle pathology on histology.	Biopsy	Japan
6	10	non-DMD	No sign of muscle pathology on histology.	Biopsy	Japan
7	14	non-DMD	No sign of muscle pathology on histology.	Biopsy	Japan
8	15	non-DMD	No sign of muscle pathology on histology.	Biopsy	Japan
9	18	non-DMD	No sign of muscle pathology on histology.	Biopsy	Japan
10	32	non-DMD	No sign of muscle pathology on histology.	Biopsy	Japan
11	2	DMD	exon66: c. 9568C>T (R3190X)	Biopsy	Japan
12	2	DMD	exon33: c.4540G>T (E1514X)	Biopsy	Japan
13	2	DMD	exon33: c.4540G>T (E1514X)	Biopsy	Japan
14	2	DMD	exon53_55del	Biopsy	Japan
15	3	DMD	exon7: c.568C>T (Q190X)	Biopsy	Japan
16	3	DMD	exon21: c. 2701G>T (G901X)	Biopsy	Japan
17	3	DMD	exon59: c.8713C>T (R2905X)	Biopsy	Japan
18	3	DMD	exon62: c.9204_9207delCAAA (N3068KfsX20)	Biopsy	Japan
19	3	DMD	exon23: c.3079G>T (G1027X)	Biopsy	Japan
20	4	DMD	exon14: c. 1663C>T (Q555X)	Biopsy	Japan
21	4	DMD	exon20_29del	Biopsy	Japan
22	5	DMD	exon10: c.1087C>T (Q363X)	Biopsy	Japan
23	5	DMD	exon41: c.5867G>A (W1956X)	Biopsy	Japan
24	5	DMD	exon8_17dup	Biopsy	Japan
25	6	DMD	exon41: c.5758C>T (Q1920X)	Biopsy	Japan
26	6	DMD	exon59: c.8692C>T (Q2898X)	Biopsy	Japan
27	8	DMD	exon13: c.1533_1536delTCAC (H512WfsX4)	Biopsy	Japan
28	9	DMD	exon20: c.2435G>A (W812X)	Biopsy	Japan
29	9	DMD	exon48_50del	Biopsy	Japan
30	10	DMD	Intron6: c.530+1G>A	Biopsy	Egypt
31	10	DMD	Decreased immunoreactivity to dystrophin. Typical clinical signs of DMD.	Biopsy	Japan
32	10	DMD	Decreased immunoreactivity to dystrophin. Typical clinical signs of DMD.	Biopsy	Japan
33	10	DMD	exon17_44dup	Biopsy	Japan
34	11	DMD	Decreased immunoreactivity to dystrophin. Typical clinical signs of DMD.	Biopsy	Japan
35	11	DMD	Decreased immunoreactivity to dystrophin. Typical clinical signs of DMD.	Biopsy	Myanmar
36	12	DMD	Decreased immunoreactivity to dystrophin. Typical clinical signs of DMD.	Biopsy	Japan
37	12	DMD	Decreased immunoreactivity to dystrophin. Typical clinical signs of DMD.	Biopsy	Japan
38	13	DMD	Decreased immunoreactivity to dystrophin. Typical clinical signs of DMD.	Biopsy	Japan
39	14	DMD	Decreased immunoreactivity to dystrophin. Typical clinical signs of DMD.	Biopsy	Japan
40	14	DMD	Decreased immunoreactivity to dystrophin. Typical clinical signs of DMD.	Biopsy	Japan
41	20	DMD	exon12, 13, 17 19del	Autopsy	Japan
42	23	DMD	Decreased immunoreactivity to dystrophin. Typical clinical signs of DMD.	Autopsy	Japan
43	26	DMD	exon43: c. 6283C>T (R2095X)	Autopsy	Japan
44	33	DMD	Decreased immunoreactivity to dystrophin. Typical clinical signs of DMD.	Autopsy	Japan

Supplementary Figure 1

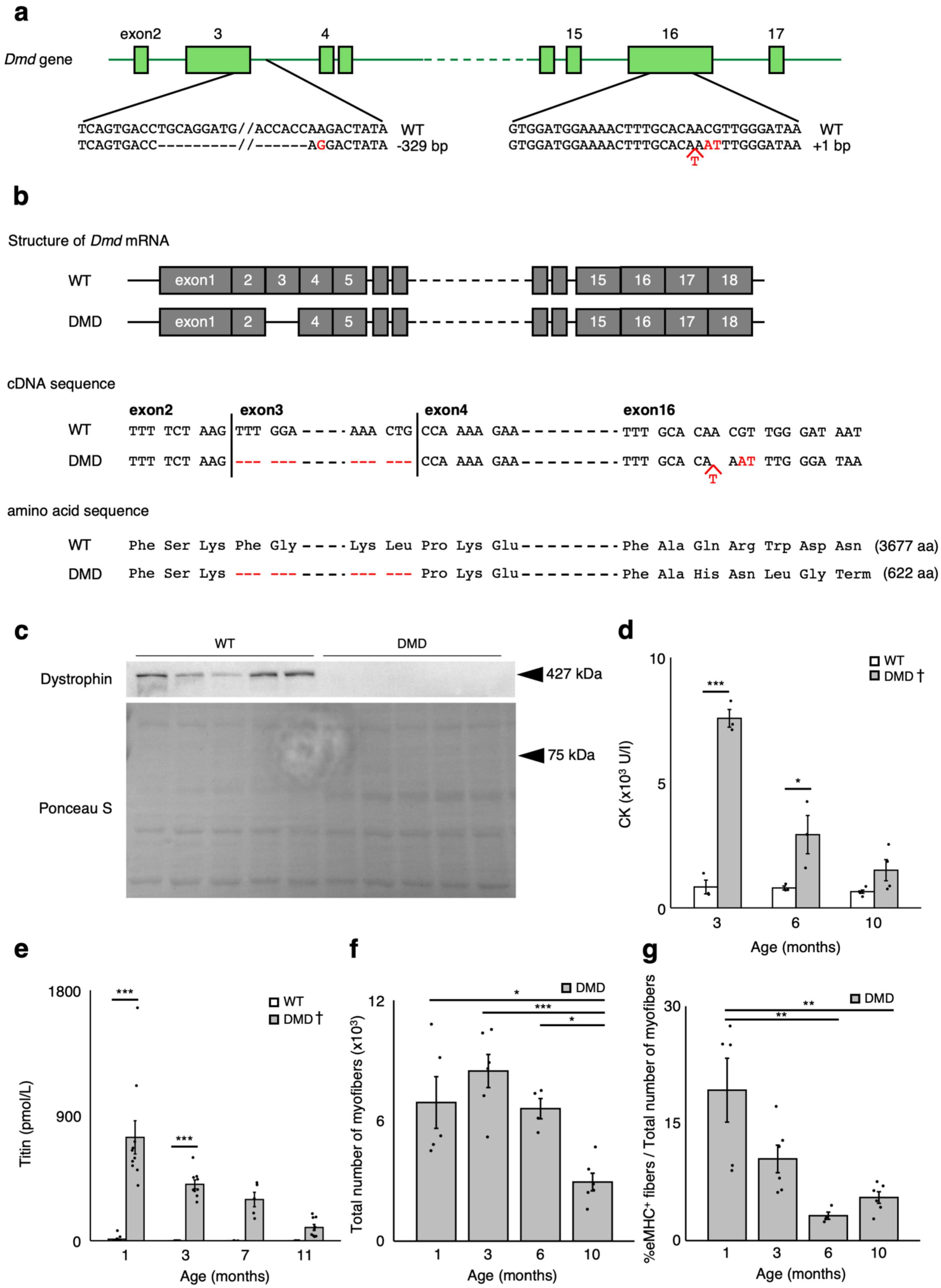
a
exon2
3
4
15
16
17
Dmd gene
TCAGTGACCTGCAGGATG//ACCACCAAGACTATA
TCAGTGACC---------//------AGGACTATA
WT
-329 bp
GTGGATGGAAAACTTTGCACAACGTTGGGATAA
GTGGATGGAAAACTTTGCACAAATTTGGGATAA
T
WT
+1 bp
b
Structure of Dmd mRNA
WT
DMD
exon1
2
3
4
5
15
16
17
18
cDNA sequence
exon2
exon3
exon4
exon16
WT TTT TCT AAG TTT GGA ---- AAA CTG CCA AAA GAA --------- TTT GCA CAA CGT TGG GAT AAT
DMD TTT TCT AAG --- --- ---- --- --- CCA AAA GAA --------- TTT GCA CA AAT TTG GGA TAA
T
amino acid sequence
WT Phe Ser Lys Phe Gly ---- Lys Leu Pro Lys Glu --------- Phe Ala Gln Arg Trp Asp Asn (3677 aa)
DMD Phe Ser Lys --- --- ---- --- --- Pro Lys Glu --------- Phe Ala His Asn Leu Gly Term (622 aa)
c
WT
DMD
Dystrophin
427 kDa
75 kDa
Ponceau S
d
CK (x10³ U/l)
10
5
0
3
6
10
Age (months)
WT
DMD †
***
*
e
Titin (pmol/L)
1800
900
0
1
3
7
11
Age (months)
WT
DMD †
***
***
f
Total number of myofibers (x10³)
12
6
0
1
3
6
10
Age (months)
DMD
*
***
*
g
%eMHC⁺ fibers / Total number of myofibers
30
15
0
1
3
6
10
Age (months)
DMD
**
**

Supplementary Figure 1. *Dmd* gene mutation pattern and elevated biochemical markers of muscular damage in DMD rats.**(a)** Sequence of the *Dmd* gene in DMD rats. **(b)** Structure and cDNA sequence of *Dmd* mRNA in WT and DMD rats. Deletion of splice site in intron2 as indicated in **(a)** leads to exon3 skipping in DMD rats. One base insertion in exon16 causes the generation of a stop codon as indicated in the amino acid sequence below. Term = stop codon. aa = amino acids. **(c)** Immunoblotting analysis of dystrophin expression using an antibody to detect dystrophin C-terminus in 3 month-old WT and DMD rats. Full-length blots are presented in Supplementary Figure 6b. **(d)** Serum creatine kinase (CK) activity in WT and DMD rats (WT: n=3, 3, 5; DMD: n=3, 3, 4). **(e)** Urinary titin concentrations in WT and DMD rats (WT: n=10, 9, 5, 9; DMD: n=10, 9, 5, 9). **(f,g)** **(f)** Total number of myofibers was counted in the TA muscle sections, and **(g)** the number of $eMHC^+$ myofibers was divided by the total number of myofibers. Data are expressed as means+SEM, and were compared by Tukey Kramer's test. For **(d)** and **(e)**, the result of statistical comparison only between the genotypes at each indicated ages was displayed. When a significant age-related difference was observed by the Tukey-Kramer's test, the † mark was added beside the legend of the graph. *p<0.05. **p<0.01. ***p<0.001.

.

Supplementary Figure 2

a

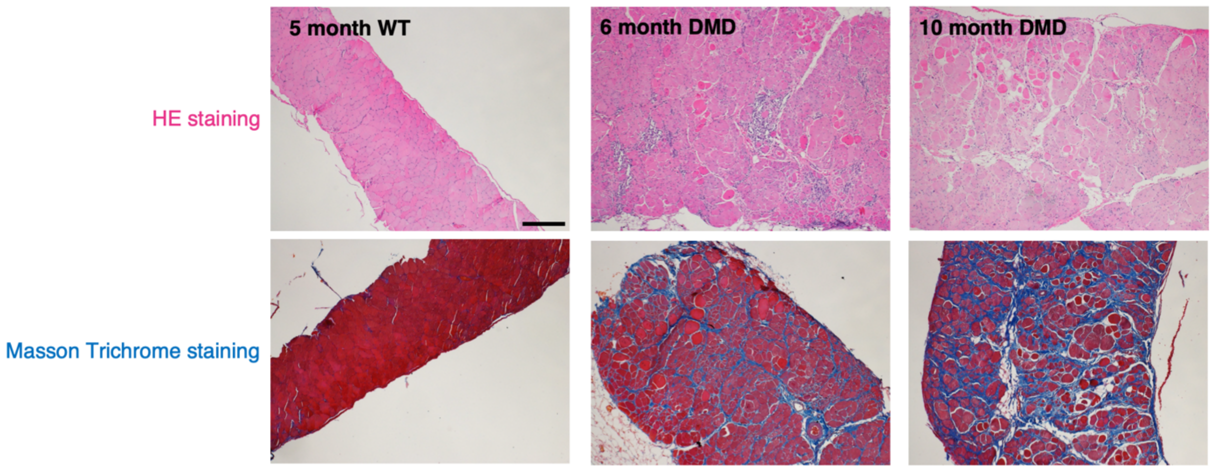
5 month WT
6 month DMD
10 month DMD
HE staining
Masson Trichrome staining

b

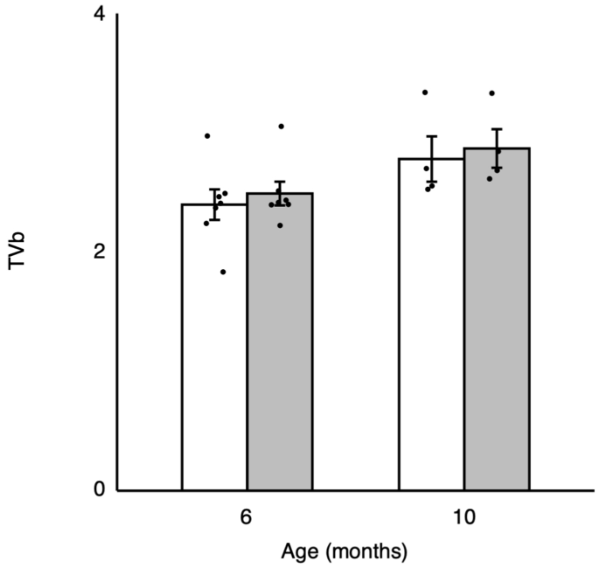
4
2
0
TVb
6
10
Age (months)

Supplementary Figure 2. Progressive exacerbation of histopathology of the diaphragm in DMD rats without functional decline. (a) Histological analysis of diaphragms by HE staining (upper panel) and Masson Trichrome staining (lower panel) from 5 to 10 month-old WT and DMD rats. Scale bar = 250 μm. **(b)** Tidal volume breathing (TVb) was measured as an indicator of respiratory function in WT and DMD rats aged 6 and 9 months (WT: n=7, 4; DMD: n=7, 4). Data are expressed as means+SEM. The p value was determined by unpaired student's *t*-test.

Supplementary Figure 3

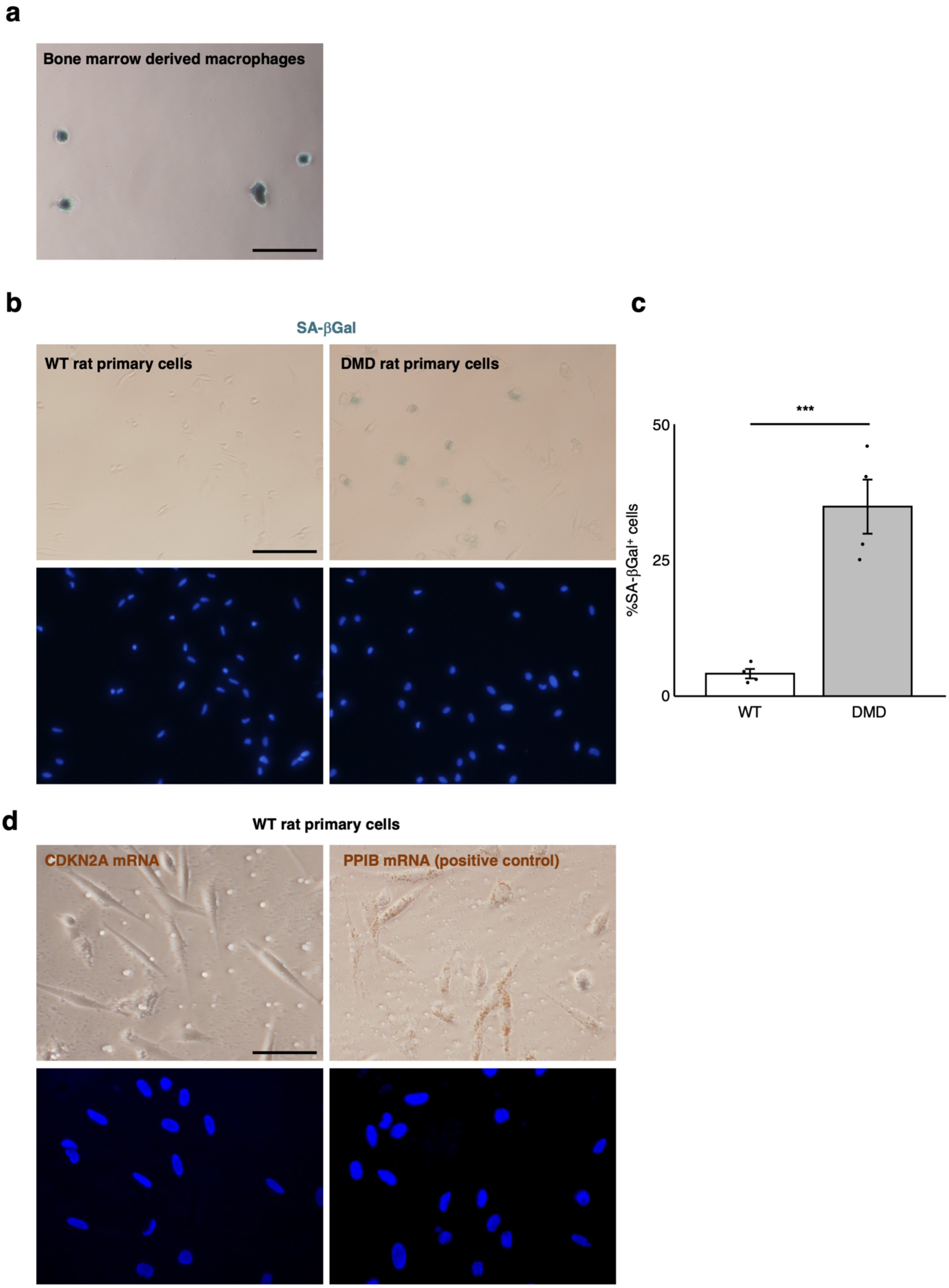
a
Bone marrow derived macrophages
b
SA-βGal
WT rat primary cells
DMD rat primary cells
c
***
50
25
0
%SA-βGal+ cells
WT
DMD
d
WT rat primary cells
CDKN2A mRNA
PPIB mRNA (positive control)

Supplementary Figure 3. Senescence induction in DMD rats. (a) SA-βGal staining was performed in Bone marrow derived macrophages. Almost all the macrophages were SA-βGal$^+$. Scale bar = 200 mm. **(b)** CD45$^+$ cells were MACS-depleted, and SA-βGal staining was performed in 3 months old WT and DMD rat skeletal muscle primary cells. Scale bar = 200 mm. **(c)** Quantification of SA-βGal$^+$ cells per field (WT: n=4, DMD: n=4). **(d)** In situ hybridization of CDKN2A mRNA using RNAscope on skeletal muscle primary cells from 3-month-old WT rats. Peptidyl-prolyl cis-trans isomerase B (PPIB) mRNA was used for the positive control of in situ hybridization. Scale bar = 100 mm.Data are expressed as means+SEM, and were compared with *t*-test. ***p<0.001.

Supplementary Figure 4

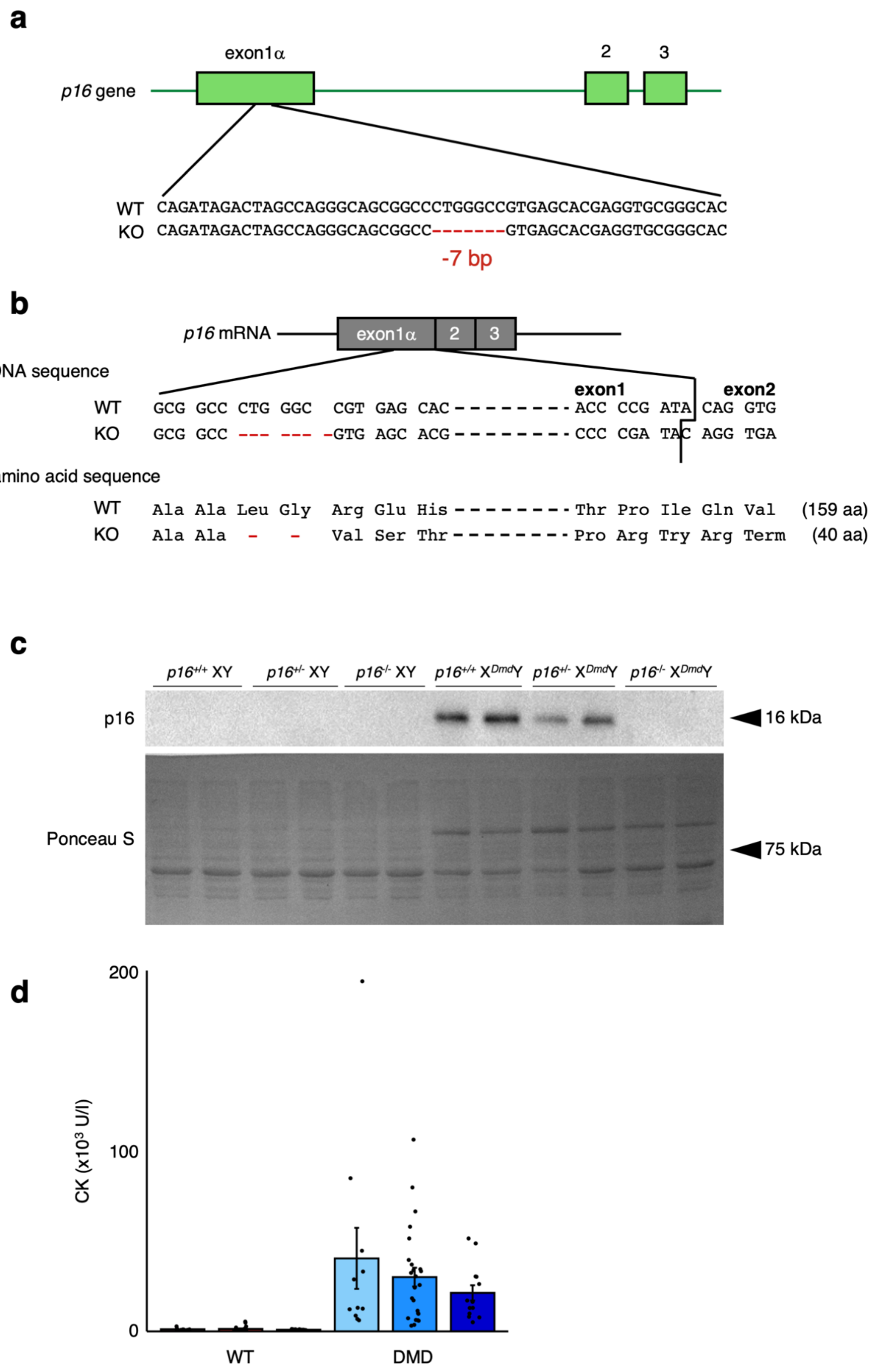
a
exon1α
2
3
p16 gene
WT CAGATAGACTAGCCAGGGCAGCGGCCCTGGGCCGTGAGCACGAGGTGCGGGCAC
KO CAGATAGACTAGCCAGGGCAGCGGCC-------GTGAGCACGAGGTGCGGGCAC
-7 bp
b
p16 mRNA
exon1α
2
3
cDNA sequence
exon1
exon2
WT GCG GCC CTG GGC CGT GAG CAC --------- ACC CCG ATA CAG GTG
KO GCG GCC --- --- -GTG AGC ACG --------- CCC CGA TAC AGG TGA
amino acid sequence
WT Ala Ala Leu Gly Arg Glu His --------- Thr Pro Ile Gln Val (159 aa)
KO Ala Ala - - Val Ser Thr --------- Pro Arg Try Arg Term (40 aa)
c
p16+/+ XY
p16+/- XY
p16-/- XY
p16+/+ XDmdY
p16+/- XDmdY
p16-/- XDmdY
p16
16 kDa
Ponceau S
75 kDa
d
200
100
0
CK (x10³ U/l)
WT
DMD

Supplementary Figure 4. *p16* gene mutation pattern and elevated biochemical markers of muscular damage in dKO rats. **(a)** Sequence of the *p16* gene in DMD rats. **(b)** cDNA sequence of *p16* mRNA in WT and DMD rats. The seven base deletions in exon1α results in a stop codon, as indicated in the amino acid sequence below. Term = stop codon. aa = amino acids. **(c)** Immunoblotting analysis of p16 expression in 9 month-old $p16^{+/+}$, $p16^{+/-}$, and $p16^{-/-}$ background WT and DMD rats. Full-length blots are presented in Supplementary Figure 6c. **(d)** Serum creatine kinase (CK) activity in 9 month-old $p16^{+/+}$, $p16^{+/-}$, and $p16^{-/-}$ background WT and DMD rats (n=9, 22, 17, 11, 25, 13). Data are compared by Tukey Kramer's test. Tukey Kramer's test was performed only between WT background groups, or between DMD background groups. Data are expressed as means+SEM.

Supplementary Figure 5

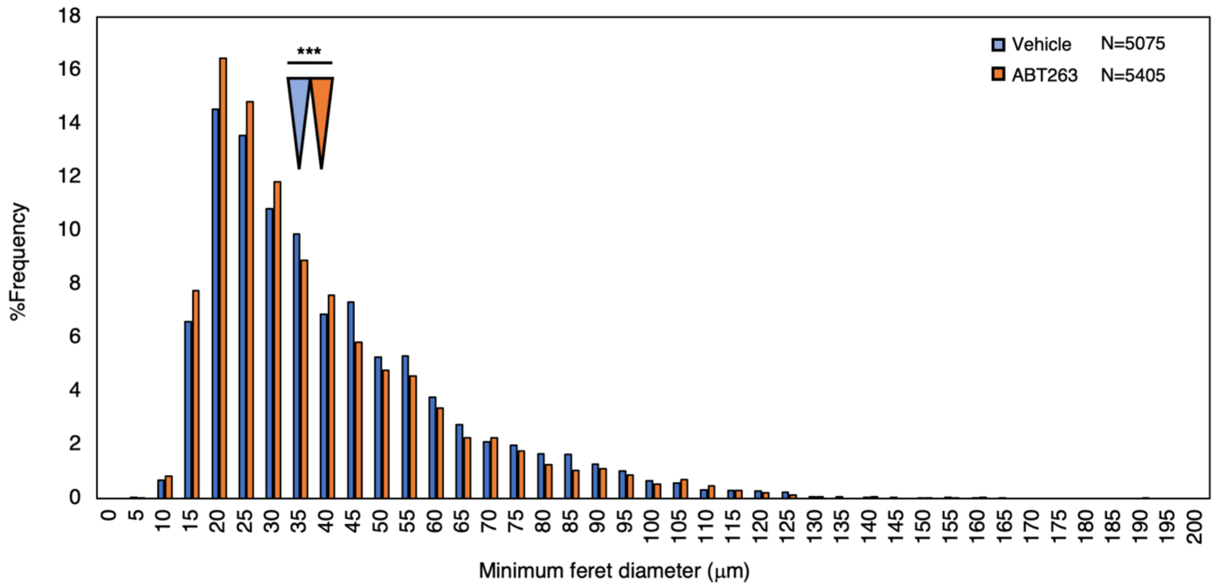

***
Vehicle N=5075
ABT263 N=5405
%Frequency
0
2
4
6
8
10
12
14
16
18
0 5 10 15 20 25 30 35 40 45 50 55 60 65 70 75 80 85 90 95 100 105 110 115 120 125 130 135 140 145 150 155 160 165 170 175 180 185 190 195 200
Minimum feret diameter (µm)

Supplementary Figure 5. ABT263 partially restores myofiber size. The size of each fiber was determined using CellProfiler software (Vehicle: n=5075, ABT263: n=5405). Arrowheads indicate the median value of myofiber size. Data are expressed as histogram, and median values were compared with Wilcoxon rank sum test. ***p<0.001.

Supplementary Figure 6

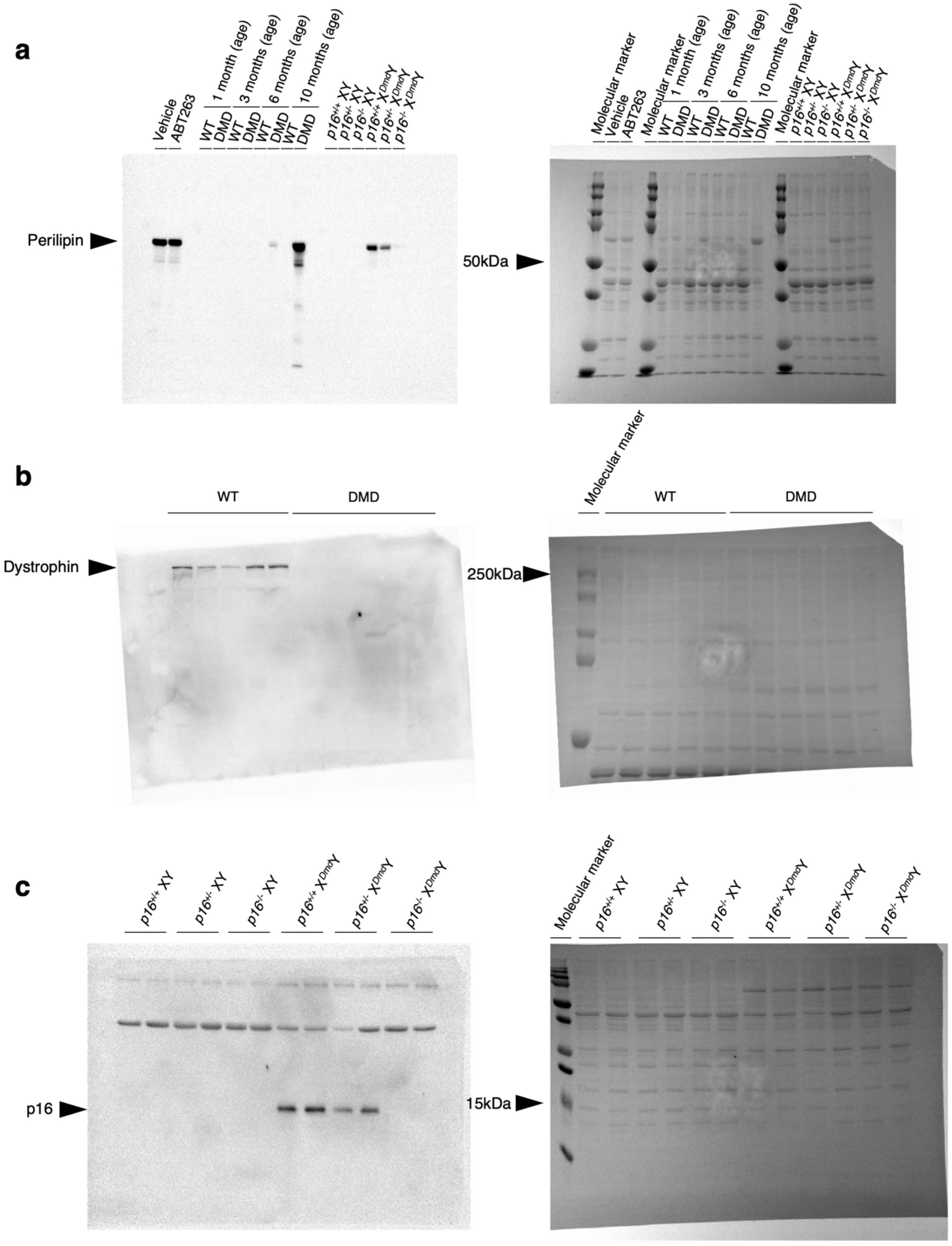
a
Vehicle
ABT263
1 month (age)
3 months (age)
6 months (age)
10 months (age)
WT
DMD
p16+/+ XY
p16+/- XY
p16-/- XY
p16+/+ XDmdY
p16+/- XDmdY
p16-/- XDmdY
Perilipin
Molecular marker
50kDa
b
WT
DMD
Dystrophin
Molecular marker
250kDa
c
p16+/+ XY
p16+/- XY
p16-/- XY
p16+/+ XDmdY
p16+/- XDmdY
p16-/- XDmdY
p16
Molecular marker
15kDa

Supplementary Figure 6. Full-length images of blots. **(a)** Full-length blots of perilipin (left panel) and corresponding Ponceau staining (right panel). **(b)** Full-length blots of dystrophin (left panel) and corresponding Ponceau staining (right panel). **(c)** Full-length blots of p16 (left panel) and corresponding Ponceau staining (right panel).
